# Reducing Small Molecule Adsorption in a PDMS-Based Microphysiological System of the Female Reproductive Tract via Parylene‑C Coating to Improve Mechanistic Studies

**DOI:** 10.1021/acsami.5c20917

**Published:** 2026-01-06

**Authors:** Rahul Cherukuri, Sungjin Kim, Haley L. Moyer, Hayley Jesse, Po Yi Lam, Lauren S. Richardson, Ananth Kumar Kammala, Ramkumar Menon, Ivan Rusyn, Arum Han

**Affiliations:** † Department of Electrical and Computer Engineering, 14736 Texas A&M University, College Station, Texas 77840, United States; ‡ Department of Veterinary Physiology and Pharmacology, College of Veterinary Medicine and Biomedical Sciences, 2655 Texas A&M University, College Station, Texas 77840, United States; § Division of Basic Science and Translational Research, Department of Obstetrics & Gynecology, 12338 The University of Texas Medical Branch at Galveston, Galveston, Texas 77555, United States; ∥ Department of Biomedical Engineering, Texas A&M University, College Station, Texas 77840, United States; ⊥ Department of Chemical Engineering, Texas A&M University, College Station, Texas 77840, United States

**Keywords:** drug screening, molecular adsorption, polydimethylsiloxane (PDMS) surface modification, Parylene coating, microphysiological system, organ-on-chip

## Abstract

Microphysiological systems (MPS) have been extensively developed in the past decade and are now used in mechanistic studies, as well as in drug and chemical toxicity testing. The utility of MPS for studies of a broad range of molecules is becoming ever more important. Many MPS models utilize polydimethylsiloxane (PDMS) as the material of choice. Despite its advantages, including biocompatibility, optical transparency, and gas permeability, PDMS exhibits significant molecular adsorption due to its hydrophobic surface properties, which is a well-known phenomenon. Although some MPS can be made from low-adsorbance materials, not all models can easily transition away from PDMS. Here, we investigated the potential of Parylene-C, a widely used coating for medical devices, for surface modification of a PDMS-based feto-maternal interface (FMi) MPS device. The impact of this coating on molecular adsorption was tested with five different chemicals (four drugs and one environmental pollutant) using a two-chambered microchannel-interconnected MPS device. We showed that the Parylene-C coating did not obstruct the microchannels, allowed chemical diffusion between chambers, did not compromise cell viability, and minimized molecular adsorption. The beneficial effect of Parylene-C coating was most prominent for highly adsorbed drugs (lipophilic)—celicoxib and tamoxifen—while the low-adsorbed compounds (amphiphilic or hydrophilic) like aspirin, sofosbuvir, and perfluorooctanoic acid were unaffected. Importantly, by limiting molecular adsorption, we were able to demonstrate chemical effects in the FMi MPS device even when using highly PDMS-adsorbed compounds. Collectively, our results demonstrate an easily adoptable strategy to increase the toxicological utility of PDMS-made MPS devices by modifying surface properties and adsorption behavior through the use of a Parylene-C coating.

## Graphical abstract

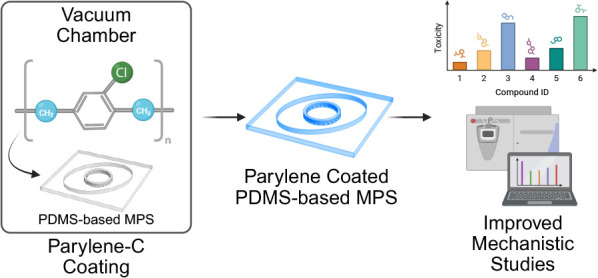

## 1 Introduction

Microphysiological systems (MPS), also known as organ-on-chip, have been extensively developed in the past decade to better mimic the structure, physiology, and responses of *in vivo* tissue/organ systems in *in vitro* culture. There is currently significant interest in the use of MPS as alternative methods for toxicity testing in many different application areas such as drug development and discovery, where it takes significant time and resources to bring a new drug to market,^1,2^ as well as assessment of the potential toxicity of industrial and household chemicals. MPS leverage various microfabrication technologies to create microstructures, in combination with cells, to replicate *in vivo*-like tissue structures and functions. Many different MPS devices have been successfully applied for disease modeling,^3–5^ mechanistic studies,^6,7^ and drug/toxicant testing.^4,8–10^ MPS offer several key advantages, including miniaturization,^11^ the ability to mimic complex tissue and organ architectures,^12^ real-time characterization,^13^ and test automation.^7,14^ As these systems mature from proof-of-concept studies to more routine use, the selection of device materials that are compatible with a wide range of physicochemical properties of drugs and chemicals becomes an important consideration. Polydimethylsiloxane (PDMS) has been extensively utilized as a building material for MPS devices, especially during prototyping stages, because it provides favorable biocompatibility, optical properties for microscopy,^15^ flexibility in medium-throughput fabrication,^16^ and gas permeability for cell culture.^17^ However, PDMS also displays high hydrophobicity that leads to undesired molecular adsorption of lipophilic compounds,^18–20^ which may impact the bioavailability of chemicals in cell culture studies,^16,19,21^ leading to gross underestimation of effective drug/chemical doses cells experience. Despite these challenges, the use of PDMS-based MPS devices continues to be popular due to the limited alternatives that materials such as plastic cannot fully overcome. Thus, to mitigate molecular adsorption to hydrophobic PDMS surfaces while retaining the benefits of using PDMS, several approaches have been proposed, including UV (ultraviolet) treatment,^16^ polyelectrolyte multilayer (PEM) application,^16,22^ silanization,^23^ and (co)­polymer addition.^24^ These approaches demonstrated the successful hydrophilization of PDMS surfaces; however, their routine use in MPS applications is still limited. For example, unstable chemical additives can lead to issues such as reduced transparency, increased contact angle, and inadequate cross-linking,^16^ while also failing to achieve uniform coating within confined geometries.^25,26^ While many MPS devices indeed have moved on to hard plastic materials commonly used for cell culture, such as polycarbonate (PC),^16^ polystyrene (PS),^27^ cyclic olefin copolymer,^28,29^ or other materials such as Flexdym,^30,31^ these materials still have issues ranging from low gas permeability^32^ to added complexity in device fabrication.^32,33^

Parylene coating is a standardized and highly reproducible chemical vapor deposition method that has been broadly utilized in many industries, including transportation,^34^ aerospace,^35^ electronics,^36,37^ semiconductors,^38,39^ and medical devices.^40,41^ It has high biocompatibility, thermal stability, and resistance to moisture, chemicals, and physical damage.^42,43^ Among the three types of Parylene conformal coatings (N, C, and D type), Parylene-C is particularly advantageous in biomedical applications as it forms a thin, conformal, and pinhole-free protective layer that enhances material stability and minimizes molecular interactions. While Parylene-N provides good biocompatibility, its lower barrier integrity and higher permeability make it less suitable for adsorption-sensitive studies. Parylene-D, despite its enhanced chemical resistance, has been reported to be less biocompatible than Parylene-C and thus has been far less commonly used for biomedical and cell-based applications.^44–47^ Notably, Parylene-C is a US Pharmacopeial Convention (USP) class IV material that has been approved by the Food and Drug Administration (FDA). Therefore, it is suitable for biomedical devices to reduce molecular adsorption and has been utilized in microfluidic devices for mammalian cell culture,^48,49^ enhanced polymerase chain reaction (PCR) performance,^50^ and to minimize moisture loss.^51^ For example, Wang et al. successfully utilized a Parylene-C-coated PDMS well to investigate the cellular proliferation as well as autophagy of breast cancer cells (MCF7 cell) and their responses to the drug tamoxifen.^52^ This study demonstrated that tamoxifen-induced cytotoxicity was comparable between Parylene-C-coated PDMS culture wells and polystyrene (PS) cell culture plates. However, the Parylene-C coating has not been widely utilized in more complex MPS systems.

In this study, we investigated the use of Parylene-C coating on a PDMS-based two-compartment feto-maternal interface (FMi) MPS model and conducted molecular adsorption and kinetic studies with four drug compounds (lipophilic drugs celecoxib and tamoxifen, hydrophilic drug sofosbuvir, and amphiphilic drug aspirin) as well as one environmental chemical, perfluorooctanoic acid (PFOA, amphiphilic) that have different molecular and PDMS adsorption properties. The impact of the Parylene-C coating was assessed based on how these chemicals affect the fetal and maternal cells cultured in the MPS model. Our findings demonstrate the potential of Parylene-C coating as a broadly applicable surface modification method for PDMS-based MPS devices without the need for complex modification of the microfabrication processes, facilitating more accurate and reproducible drug and chemical testing using MPS devices.

## 2 Methods

### 2.1 MPS Device Fabrication

The two-chamber female reproductive tract MPS model that was previously developed and utilized for many applications, such as recreating amnion membrane^5^ and choriodecidual interface,^53^ was used here as the base MPS model to test the effectiveness of Parylene-C coating in minimizing molecular adsorption. The device was fabricated in PDMS using a two-step photolithography and soft lithography process, as previously described.^5^ In brief, the master mold was created on a 4 in. silicon substrate using two layers of photosensitive epoxy (SU-8; MicroChem, Westborough, USA), each with a different thickness. The first layer, forming 5 μm high microchannels, was fabricated by spin-coating SU-8 3005 at 3,400 rpm for 45 s, followed by soft baking at 95 °C for 2 min. The layer was then UV-exposed through a photomask, postexposure baked at 65 °C for 1 min, and then at 95 °C for 2 min. The second layer, creating the 500 μm thick cell culture chambers, was spin-coated with SU-8 2100 at 1,000 rpm for 45 s, soft baked at 95 °C for 1 h, UV-exposed through a second photomask, and postexposure baked at 65 °C for 5 min, followed by another bake at 95 °C for 15 min. To ensure proper SU-8 structures, the mold was immersed in edge-bead removal solution for 15 min, rinsed with isopropyl alcohol (IPA) and deionized (DI) water, and then coated with (tridecafluoro-1,1,2,2-tetrahydrooctyl) trichlorosilane (United Chemical Technologies, Bristol, PA, USA) to facilitate the release of PDMS. PDMS molds were prepared by pouring a 10:1 mixture of PDMS prepolymer (Sylgard 184; DowDuPont, Midland, USA) onto the master mold, curing at 75 °C for 60 min. Reservoirs for the culture medium were punched using a 5 mm diameter punch bit (Syneo, Angleton, USA). The PDMS molds were then coated with Parylene-C (see Section 2.2 for details) and treated with oxygen plasma (Harrick Plasma, Ithaca, USA) for 120 s to improve bonding to glass substrates and enhance hydrophilicity for easier cell and medium loading.

### 2.2 Parylene-C Coating

The PDMS molds were coated with an ∼2 μm thick Parylene-C layer using 2.5 g of dichloro-di-*p*-xylylene (DPX-C) dimer (Specialty Coating Systems, Indianapolis, USA). The Parylene deposition process took around 1.5 h using the Labcoter 2 Parylene deposition system (PDS 2010, Specialty Coating Systems, Indianapolis, USA), following previously established protocols.^9^ During this process, the PDMS molds (taped on the bottom surface to prevent Parylene deposition on the bonding interface) were kept on a rotating platform within the vacuum chamber of the Parylene coater. After deposition, the tape was removed from the bottom surface to plasma bond the bare PDMS layer to a glass substrate.

### 2.3 Testing of Compound Transfer between Chambers

Lipopolysaccharide (LPS) conjugated with fluorescein isothiocyanate (100 μg/mL LPS-FITC; F3665, Millipore Sigma, Burlington, USA), an amphiphilic compound, and 3000 Da (kDa) fluorescent dextran (20 μg/mL 3 kDa Dextran – Texas Red; D3329, Thermo Fisher Scientific, Waltham, USA), a highly hydrophilic compound, were utilized to evaluate transport dynamics from the outer culture chamber (200 μL) to the inner culture chamber (65 μL) through the microchannel array, since both molecules have been previously shown not to adsorb to PDMS in studies with the two-chamber MPS device.^5^ Bare (uncoated) and Parylene-C-coated devices were tested and compared. Following the loading of LPS-FITC or 3 kDa dextran to the outer chambers, 50 μL of samples were collected from both outer and inner chambers at 24, 48, and 72 h time points. Fluorescence measurements were conducted using a microplate reader (Biotek Synergy H1, Agilent, Santa Clara, USA) with excitation set at 495 nm and emission at 520 nm for LPS-FITC, and excitation set at 595 nm and emission at 615 nm for 3 kDa dextran.

### 2.4 Chemical Adsorption Testing

To evaluate and compare the adsorption of chemicals in uncoated versus Parylene-C-coated PDMS-based MPS devices, a 100 μM solution of each compound in cell culture media was introduced into both chambers of the devices. Five compounds were tested in this study: aspirin (A5376-100G, Millipore Sigma, Burlington, USA), celecoxib (SML3031-50MG, Millipore Sigma), sofosbuvir (AMBH2D6FB19C-100MG, Millipore Sigma), tamoxifen (T5648-1G, Sigma-Aldrich, St. Louis, USA) as representative drugs with different properties, and PFOA (171468-5G, Sigma-Aldrich), a persistent environmental pollutant. After 48 h of incubation with the molecules, the medium from each compartment was collected and stored at −80 °C until later analysis. Compound adsorption was quantified using mass spectrometry (see Section 2.10 for details). Controls included medium containing known compound concentrations and blank medium in a 96-well plate.

### 2.5 Cell Culture

Human maternal decidual cells (DECs) and human fetal membrane amnion epithelial cells (AECs) were isolated and immortalized following protocols detailed in our previous studies.^54,55^ DECs were cultured in Dulbecco’s Modified Eagle’s Medium (DMEM) combined with Ham’s F12 nutrient mixture (1:1), supplemented with 5% heat-inactivated fetal bovine serum and antibiotics: penicillin (100 IU/mL), streptomycin (100 μg/mL), and amphotericin B (2.5 μg/mL). AECs were grown in complete keratinocyte serum-free medium (KSFM), which is optimized for epithelial cell growth and enriched with 0.1 ng/mL human recombinant epidermal growth factor, 30 μg/mL bovine pituitary extract, and 0.5 mg/mL primocin (ant-pm-1, InvivoGen). All supplements were freshly added to the medium before use.

### 2.6 Cell Seeding and Culture in the MPS Devices

Before use, the MPS devices (both uncoated and coated) were sterilized with 70% ethanol for 15 min, followed by three washes with PBS as described previously.^5,8,9^ They were then rinsed three times with a complete DMEM-F12 medium to prepare for cell seeding. DECs and AECs were seeded into MPS devices and 96-well plates. For the MPS devices, 65,000 DECs in 160 μL of DEC-specific culture medium were introduced into the outer compartment (1.42 cm^2^ surface area), while 30,000 AECs in 65 μL of AEC-specific culture medium were seeded into the inner compartment (0.283 cm^2^ surface area). For the 96-well plates (0.32 cm^2^ surface area per well), 15,000 DECs in 75 μL and 34,000 AECs in 75 μL of cell-specific culture medium were seeded. These cell densities ensured that the surface area-to-cell density ratio and medium volume remained consistent between the MPS devices and the 96-well plate experiments. The devices and plates were incubated at 37 °C with 5% CO_2_ for 24 h to allow for cells to adhere before any treatments or assays were performed.

### 2.7 Chemical Treatments for Toxicity Assessment

Initial concentration–response assays were conducted to determine the cytotoxic effects and obtain the point of departure curves for tamoxifen and celecoxib on DECs and AECs using six concentrations: 0.3, 1, 3, 10, 30, and 100 μM. Next, the devices (both chambers) and 96-well plates were individually treated (after an initial 24 h incubation period) with a range of compounds: aspirin, sofosbuvir, tamoxifen, and PFOA were each administered at a final concentration of 100 μM, while celecoxib was administered at varying concentrations of 50, 100, and 200 μM. The chemical stocks were prepared in 100% cell culture-grade dimethyl sulfoxide (DMSO). Final test concentrations in a cell-specific culture medium contained 0.5% DMSO. Following 48 h of incubation at 37 °C with 5% CO_2_, the supernatants were collected, and a cell cytotoxicity assay was conducted to assess the effects of molecular treatments on the cells. All experiments contained media only and 0.5% DMSO only (vehicle control) as negative controls, and 100 μM tetraoctylammonium bromide (TAB) as a positive control.

### 2.8 LPS Treatment for Assessment of Cellular Inflammation

To further assess the biocompatibility of Parylene-C as a coating material in PDMS-based MPS devices, 100 ng/mL lipopolysaccharide (LPS; L2880, Sigma-Aldrich, Burlington, VT, USA) was used to modulate inflammatory responses in both uncoated and Parylene-C-coated devices. After 24 h of cell culture in both coated and uncoated devices, LPS was introduced to the cell culture chambers, and supernatants were collected 48 h posttreatment to quantify pro-inflammatory cytokine levels (see Section 2.12 for details).

### 2.9 Cytotoxicity Assay

The AlamarBlue assay (A50101, Thermo Fisher Scientific, Waltham, USA) was used to evaluate metabolic activities within the MPS devices and 96-well plates. At the end of each time point, the supernatant in each chamber was collected for further analysis and replaced with 10% AlamarBlue reagent, diluted in cell-specific media. The devices were then incubated at 37 °C for 3 h. Fluorescence intensity was measured using a microplate reader (BioTek Synergy H1, Agilent, Santa Clara, USA) with excitation at 560 nm and emission at 590 nm. Fresh cell culture media mixed with AlamarBlue were used as a blank reference.

### 2.10 Immunocytochemistry (ICC) Staining

Immunocytochemical staining was performed on cells within the MPS devices to detect two biomarkers, vimentin (3.33 μL/mL; ab92547; Abcam, Cambridge, USA) and cytokeratin (CK)-18 (1.25 μL/mL; ab668; Abcam, Cambridge, MA, USA), to assess the native cell phenotypes (mesenchymal and epithelial, respectively). After 48 h of culture, cells were fixed overnight with 4% paraformaldehyde (PFA), permeabilized for 1 h using 0.5% Triton X, and blocked for 3 h with 3% bovine serum albumin (BSA) in PBS. Primary antibodies were applied and incubated overnight at 4 °C. Cells were then washed with PBS and incubated for 3 h in a light-protected environment with Alexa Fluor 488- and 596-conjugated secondary antibodies (ab150077 and ab150116; Abcam, Cambridge, USA), diluted 1:1,000 in 3% BSA. After additional PBS washes, cell nucleus was stained with NucBlue Live ReadyProbes Reagent (R37605, Thermo Fisher Scientific, Waltham) for 1 h. Bright-field and fluorescence microscopy were conducted using the Keyence all-in-one Fluorescence BZ-X810 microscope (at 4×, 10×, and 20× magnification) to assess cell morphology, viability, and specific cell morphology markers.

### 2.11 Mass Spectrometry

#### 2.11.1 Sample Preparation

Internal standards for each compound were prepared in 100% acetonitrile (AA22927K7, Fisher Scientific, Hampton, USA) at the appropriate concentrations and chilled. Specifically, aspirin D4 at 5 μM (18243, Cayman Chemical, Ann Arbor, USA), celecoxib D7 at 1 μM (18248, Cayman Chemical, Ann Arbor, USA), sofosbuvir D6 at 2 μM (HY-15005S1, MedChem Express, Monmouth Junction, USA), repaglinide (tamoxifen) at 1 μM (R9028-50MG, Sigma-Aldrich, St. Louis, USA), and C13 PFOA at 1 μM (MPFOA, Wellington Laboratories, Canada) were used. A standard curve was obtained through serial dilution using culture media, with concentrations (0 to 300 μM) based on the testing requirements. Fifty microliters of extracted samples were transferred to 1.5 mL centrifuge tubes, and 100 μL of chilled internal standard in 100% acetonitrile was added. The tubes were vortexed for 10 s and centrifuged at 12,000 rpm for 10 min at room temperature. The supernatant was then transferred to new centrifuge tubes and dried by using a speed vacuum. The residue was reconstituted in 50 μL of mobile phase A (HPLC-grade water with 0.1% formic acid [A998-4, Thermo Fisher, Waltham, USA] for aspirin, celecoxib, sofosbuvir, and tamoxifen; 5 mM ammonium acetate in water for PFOA) and vortexed for 10 s. The reconstituted samples were transferred to liquid chromatograph (LC)–mass spectrometry (MS) vials with inserts (4400-FIV2W, Ibis Scientific, Las Vegas, USA) and pre-slit caps (4000-064, Ibis Scientific, Las Vegas, USA), then stored at −20 °C until analysis.

#### 2.11.2 Sample Analyses

Tamoxifen samples (10 μL) were injected into a ZORBAX SSHD Eclipse Plus C18 column (3.0 mm × 50 mm, 1.8 μm, Cat. 959757-302, Agilent, Santa Clara) with a guard column (2.1 mm × 5 mm, 1.8 μm, Cat. 821725-901, Agilent, Santa Clara, USA) using a 1290 Infinity II LC (Agilent) and analyzed using a triple quad mass spectrometer (Agilent 6470A). All analyses were performed using positive electrospray ionization. Gas temperature, sheath gas temperature, and nebulizer pressure were set to 350 °C, 300 °C, and 275 kPa, respectively. Binary pump composition for the analysis of tamoxifen begins with 90% mobile phase A and 10% mobile phase B (0.1% formic acid in acetonitrile) and then increases to 25% mobile phase B at minute 1. Mobile phase B increases to 75% at minute 2 and then increases to 98% B at minute 3. Mobile phase B decreases to 25% at minute 4 and then returns to the initial conditions at minute 5. Sample run time was 5.5 min.

Aspirin, celecoxib, and sofosbuvir samples (5 μL) were injected into the same ZORBAX SSHD Eclipse Plus C18 column with a guard column using a 1290 Infinity II LC and analyzed using a triple quad mass spectrometer. Celecoxib and sofosbuvir were analyzed using positive electrospray ionization, while aspirin was analyzed with negative electrospray ionization. Gas temperature, sheath gas temperature, and nebulizer pressure were set to 350 °C, 350 °C, and 310 kPa to analyze celecoxib and sofosbuvir. For the analysis of aspirin, these parameters were set to 300 °C, 300 °C, and 275 kPa, respectively. Mobile phase composition for all samples begins with 90% mobile phase A (water containing 0.1% formic acid) and 10% mobile phase B (acetonitrile with 0.1% formic acid) until minute 1, then changes to 75% A and 25% B at minute 1. At minute 1.5, the amount of mobile phase B increases to 50%, and then increases again at minute 3 to 98% B. The gradient changes back to 50/50 at minute 4. The gradient returns to initial conditions at minute 5 and remains until the end of the sample analysis at minute 6.

PFOA samples (5 μL) were also injected into the ZORBAX SSHD Eclipse Plus C18 column with a guard column using a 1290 Infinity II LC and analyzed using a triple quad mass spectrometer. Samples were analyzed with negative electrospray ionization, and gas temperature, sheath gas temperature, and nebulizer pressure were set to 130 °C, 375 °C, and 241 kPa, respectively. The mobile phase composition begins with 70% mobile phase A (5 mM ammonium acetate in water) and 30% mobile phase B (acetonitrile) until minute 3.5, then changes to 10% A and 90% B. At minute 4.5, the amount of mobile phase B increases to 100%, The gradient changes to 60% B at minute 5. The gradient returns to the initial conditions at minute 5.5 and remains until the end of the sample analysis at minute 7.

### 2.12 Inflammatory Cytokine Markers Analysis

At each time point, culture supernatants were collected from both chambers to quantify inflammatory cytokine production. Luminex multiplex assays were conducted using antibody-coated beads (HCYTA-60K, EMD Millipore, Burlington, USA) to evaluate the inflammatory response and determine biomacromolecule adsorption. Standard curves were generated by using measurements of manufacturer-supplied recombinant protein standards. Cytokine concentrations in the samples were determined by correlating the measured signal values with the standard curves by using linear regression analysis.

### 2.13 Statistical Analyses

Graphs were generated using Prism 10 software (GraphPad Software, La Jolla, CA). Statistical analysis was performed using the Mann–Whitney U test and Student’s *t*-test, with significance defined at *p* < 0.05. The graphs represent mean intensities/concentrations with error bars indicating the standard error of the mean (SEM).

## 3 Results

### 3.1 Design and Fabrication of Surface-Modified PDMS-Based Two-Chamber MPS Device

The two-chamber MPS device is a PDMS-based model designed for the coculture of maternal cells (decidual cells, DECs) in the outer chamber and fetal cells (amnion epithelial cells, AECs) in the inner chamber.^5,53^ The outer elliptical chamber/compartment has a surface area of 1.42 cm^2^, while the inner circular compartment has a surface area of 0.283 cm^2^, with both chambers having a height of 300 μm. The outer and inner chambers are connected via an array of 24 microchannels, each measuring 5 μm in height, 30 μm in width, and 300 μm in length, enabling controlled fluid exchange and cell interaction between the two chambers. The device has two media reservoirs for the outer chamber and one reservoir for the inner chamber (Figure 1A and Figure S1). The coating process of Parylene-C begins with the vaporization of a powdered dimer under vacuum conditions at 135 °C, resulting in the formation of a dimeric gas (di *para*-xylylene chloride). This gas is then subjected to pyrolysis at 690 °C, which decomposes the dimer into its monomeric form (*para*-xylylene chloride). These monomers then flow into a vacuum chamber containing the PDMS devices to be deposited as a transparent Parylene-C film [poly­(monochloro-*para*-xylylene)] (Figure 1B).

**Figure 1:**
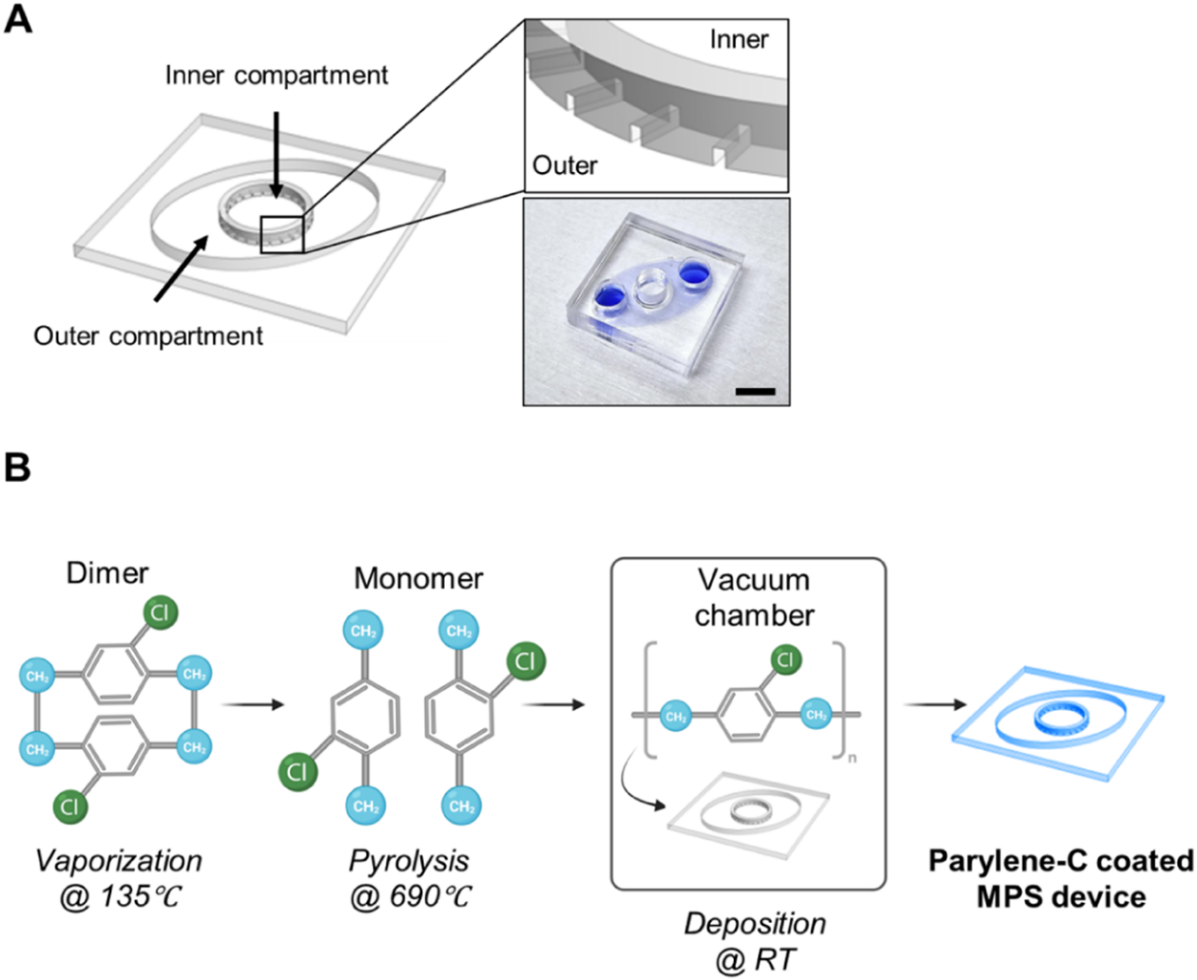
Visualization of the PDMS-based two-chamber MPS model used for testing the Parylene-C coating. A. Two-chambered MPS model consisting of a circular inner and an elliptical outer cell culture compartment connected by an array of microchannels. Image of a fabricated device filled with blue dye is also shown. Scale bar: 5 mm. B. Solid Parylene-C dimer is vaporized to monomer gas through two annealing steps and then flown into a vacuum chamber to coat the target MPS devices at room temperature. (Created with Biorender.com).

### 3.2 MPS Device Retains Its Functionalities (Compound Transfer, Cell Viability, and Biocompatibility) after Surface Modification with Parylene-C

To verify that the Parylene-C coating does not interfere with the intended functionalities of the two-chamber MPS device, we investigated compound transfer between chambers as well as the full biocompatibility of the coating.

#### 3.2.1 Compound Transfer Analysis

To evaluate the diffusion of biomolecules across the microchannels between the two cell culture chambers, LPS-FITC and 3 kDa dextran (amphiphilic and hydrophilic compounds, respectively, that do not adsorb to PDMS) were introduced into the outer chambers of both coated and uncoated devices (Figure 2A). The concentrations of these molecules in the inner and outer chambers were quantified at 24, 48, and 72 h by measuring their fluorescence by using a plate reader. Both LPS-FITC and 3 kDa dextran demonstrated similar diffusion patterns in coated and uncoated devices (Figure 2B).

**Figure 2:**
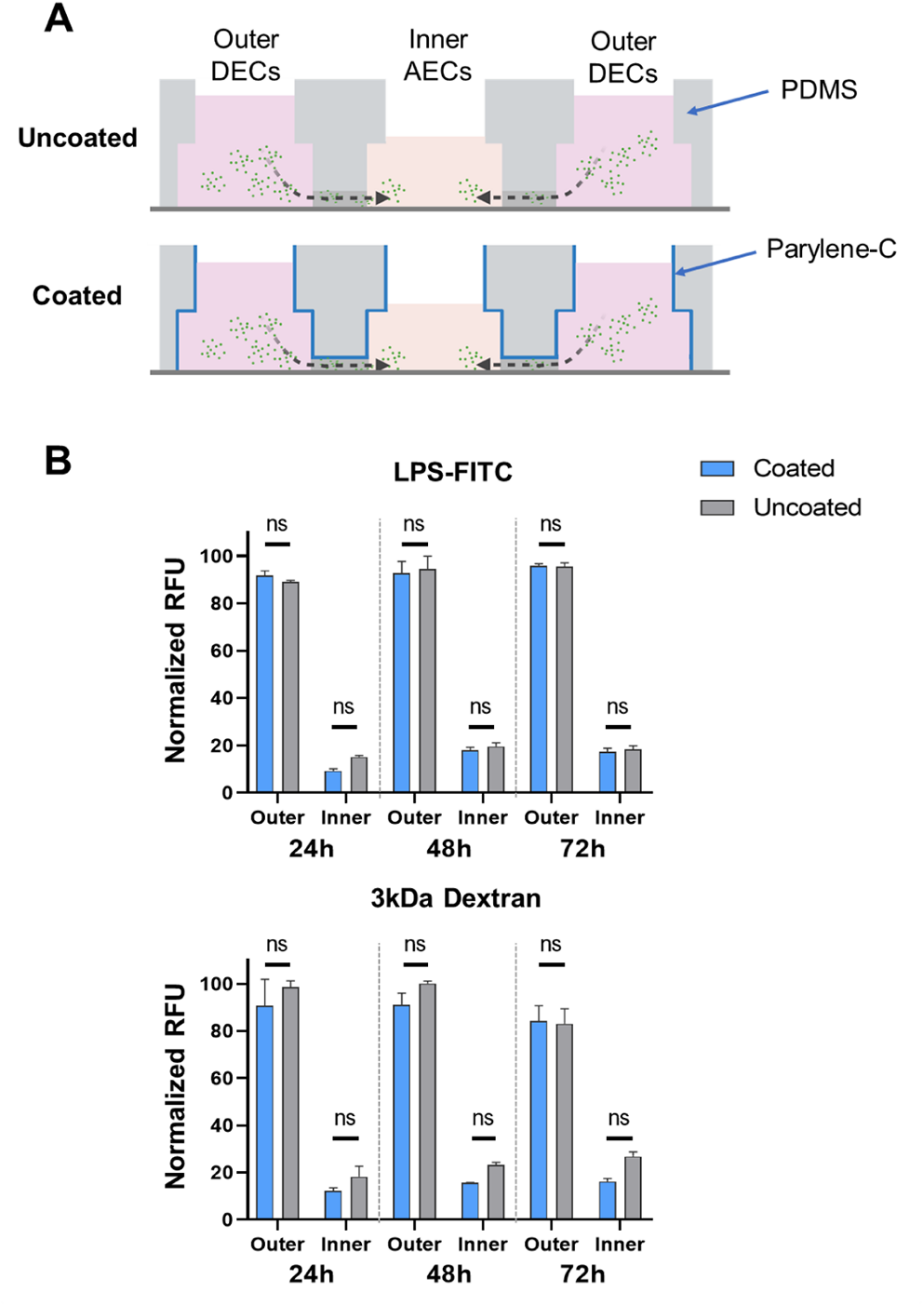
Testing the functionality (compound transfer) of the two-chamber MPS model before and after surface modification using Parylene-C. A. Visual representation of small molecule propagation in the PDMS-based MPS device with or without surface modification (coating illustrated as blue lines). B. Molecular propagation of LPS-FITC and 3 kDa Dextran in coated and uncoated MPS devices at multiple time points. Quantitative values are presented as stacked bars (mean ± SEM), and statistical analysis (coated versus uncoated devices) was conducted using the Mann–Whitney U and/or Student’s *t*-test, with significance indicated as ns—*p* > 0.05 (*n* = 3).

#### 3.2.2 Cell Viability and Cell Morphology

To ensure that the coating did not negatively impact cell viability or morphology, DECs and AECs were cultured in coated and uncoated devices and compared to the 96-well plate cultures. Cellular metabolic activity as a marker of viability was assessed using the AlamarBlue assay after 48 h. Results indicate that there are no significant differences in cell viability between coated devices [DEC: 100.3 ± 1.76%, AEC: 99.6 ± 2.89% (mean ± SEM)], uncoated devices (DEC: 102.6 ± 2.16%, AEC: 97.5 ± 2.21%), and 96-well plates (Figure 3A). ICC further confirmed that cells cultured in coated devices maintained their expected mesenchymal and epithelial morphologies, confirmed by vimentin expression in DECs and vimentin/CK-18 expression in AECs (Figure 3B). These markers confirmed the phenotypic fidelity of the cells cultured in the coated devices.^54,55^ Extended cell culture studies confirmed that cells remained viable in both coated and uncoated devices over the 7-day period (Figure S3). Correspondingly, longitudinal contact angle assessments demonstrated that the Parylene-coated PDMS sustained its hydrophilic surface characteristics, indicating preserved coating integrity throughout the extended incubation period of 7 days (Figure S2).

**Figure 3:**
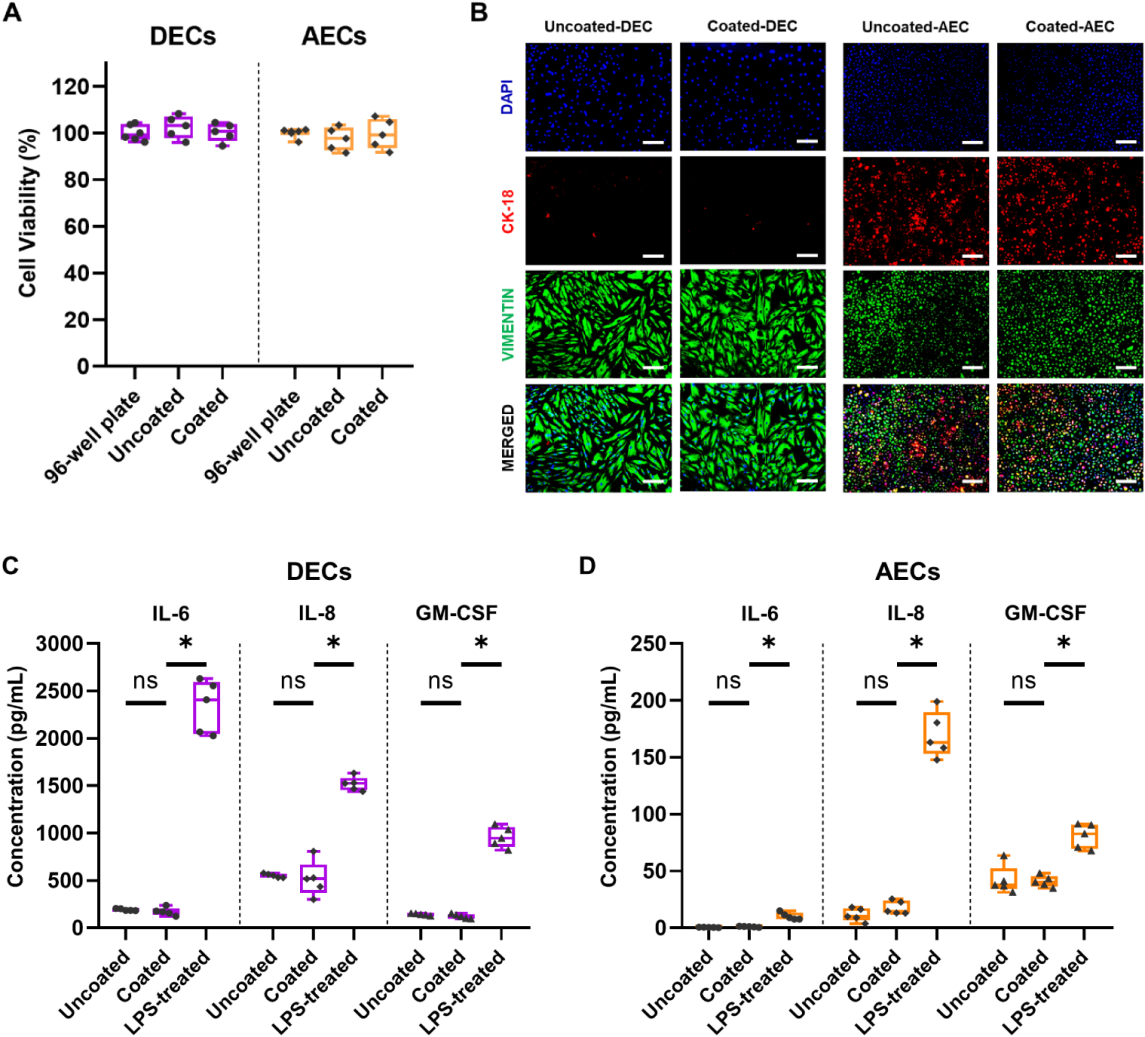
Testing the biocompatibility of the two-chamber MPS model before and after surface modification using Parylene-C. A. Cell viability of DECs and AECs within coated and uncoated MPS devices after 48 h of culture. Boxplots (median, interquartile range, max-min) are shown, and statistical analysis was performed using the Mann–Whitney U and Student’s *t*-test, where no significant differences were observed (*p* < 0.05, *n* = 5–6). B. Cell morphology was evaluated using immunocytochemistry to compare cells cultured in coated versus uncoated devices. Specific markers were analyzed to assess cellular phenotypes: CK-18 and vimentin were used to identify AECs, while vimentin alone was used to characterize the DECs. Scale bar: 20 μm. C–D, Inflammatory response of DECs (C) and AECs (D) within uncoated, coated, and LPS-treated MPS devices after 48 h of culture. Boxplots (median, interquartile range, max-min) are shown, and statistical analysis was performed using the Mann–Whitney U and Student’s *t*-test, with statistical significance indicated as ns—*p* > 0.05. *p < 0.05 (*n* = 5).

#### 3.2.3 Biocompatibility and Inflammatory Response

To evaluate the biocompatibility of the coating and ensure that it did not elicit unintended inflammation, DECs and AECs were cultured in both coated and uncoated devices and compared with LPS-treated devices serving as an inflammatory control. Cellular pro-inflammatory markers were quantified after 48 h using a Luminex multiplex assay. The results showed no significant differences in inflammatory responses between cells cultured in coated [DEC: IL6—174.1 ± 18.75, IL8—518.6 ± 83.13, GM-CSF—121.8 ± 10.67; AEC: IL6—1.09 ± 0.17, IL8—17.8 ± 2.61, GM-CSF—40.9 ± 2.21 (mean ± SEM)] and uncoated devices (DEC: IL6—193.4 ± 5.06, IL8—552.9 ± 9.39, GM-CSF—141.8 ± 4.84; AEC: IL6—0.53 ± 0.07, IL8—11.6 ± 2.61, GM-CSF—42.1 ± 5.60), whereas LPS-treated devices exhibited a marked elevation in pro-inflammatory markers (DEC: IL6—2338.9 ± 123.75, IL8— 1519.7 ± 33.24, GM-CSF—957.9 ± 48.97; AEC: IL6—10.3 ± 1.39, IL8—169.8 ± 9.01, GM-CSF—80.7 ± 4.89) (Figure 3C,D and Figure S4). These findings confirm that the Parylene-C coating is biocompatible and does not induce inflammation in the cells cultured within the coated devices. Additionally, the comparable cytokine levels between coated and uncoated devices further indicate that the coating does not differentially adsorb these biomolecules.

### 3.3 Chemical Effects on FMi Cells

Cell viability was assessed using the AlamarBlue assay to determine the cytotoxic effects of selected test compounds on DECs and AECs cultured in 96-well plate, aiming to identify appropriate dosing concentrations to be used in the MPS experiments (Figure 4 and Figure S5). Concentration–response experiments were conducted to assess the effects of tamoxifen and celecoxib on DECs and AECs using six concentrations: 0.3, 1, 3, 10, 30, and 100 μM. Tamoxifen had an IC_50_ of 20.6 μM for DECs and 6.9 μM for AECs, and Celecoxib had an IC_50_ of 44.2 μM for DECs and 42.3 μM for AECs (Figure S5). Notably, exposure to a 100 μM concentration of tamoxifen and a 200 μM concentration of celecoxib significantly reduced the viability of both DECs (tamoxifen: 0.03 ± 0.01%, celecoxib: 0.98 ± 0.01%) (Figure 4A) and AECs (tamoxifen: 0.01 ± 0.00%, celecoxib: 0.00 ± 0.00%) (Figure 4B), which will serve as good positive control drug compounds that show cellular toxicity. In contrast, 100 μM concentrations of aspirin (DECs: 103.7 ± 2.51%, AECs: 99.7 ± 1.04%), sofosbuvir (DECs: 102.2 ± 1.87%, AECs: 98.0 ± 0.98%), and PFOA (DECs: 96.0 ± 1.12%, AECs: 98.6 ± 0.82%) showed no toxic effect on cell viability and thus will serve as negative control compounds (Figure 4).

**Figure 4:**
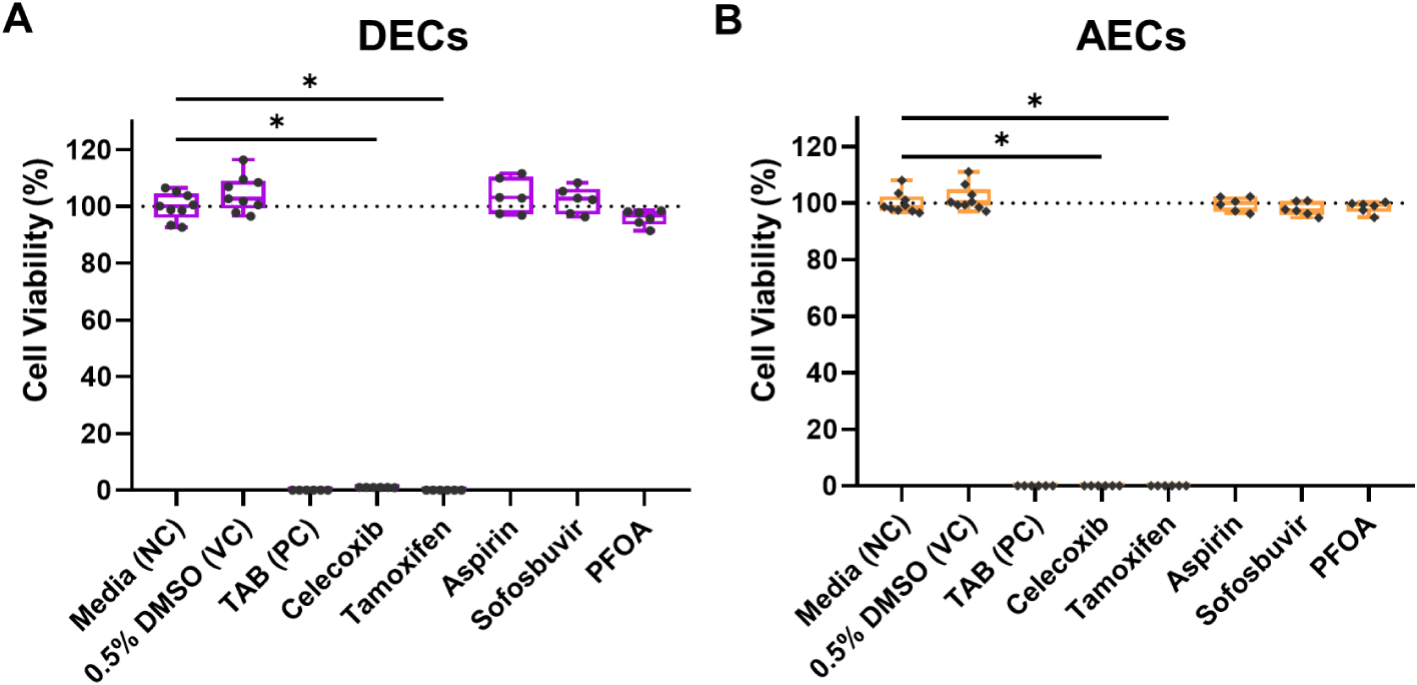
Cell viability to evaluate the effects of five different compounds (100 μM of tamoxifen, aspirin, sofosbuvir, and PFOA, 200 μM of celecoxib) on DECs (A) and AECs (B) in 2D 96-well plate experiments after 48 h of exposure. 100 μM of tetraoctylammonium bromide (TAB) served as the positive control, cell-specific culture medium as the negative control, and 0.5% DMSO as the vehicle control. Values are presented as a boxplot (median, interquartile range, max-min), and statistical analysis (compared to negative control) was conducted using the Mann–Whitney U test and Student’s *t*-test, with statistical significance indicated as *p < 0.05 (*n* = 9 for controls, *n* = 6 for test compounds).

### 3.4 Impact of Parylene-C Coating on Molecular Adsorption and Cytotoxicity of Selected Drug Compounds

To evaluate the adsorption of small molecules and drug compounds within PDMS-based MPS devices, the compounds were introduced into both Parylene-C-coated and uncoated devices, and their concentrations were measured at 48 h and normalized to baseline levels (% stock) at 0 h. In several cases, substantial molecular binding of lipophilic compounds to uncoated PDMS was observed, which can reduce compound bioavailability within the device, potentially compromising the accuracy of cytotoxicity assessments and leading to inaccurate interpretations of drug efficacy in MPS experiments. In contrast, the Parylene-C coating significantly mitigated binding, preserving compound availability for cellular exposure.

For instance, when tamoxifen was introduced into the devices, only 0.12 ± 0.003% of the stock concentration (mean ± SEM; measured concentration: < 0.5 μM) was recovered from the uncoated devices, while 23.4 ± 2.73% (measured concentration: 6.7 ± 1.75 μM) was recovered from the coated devices (Figure 5A). Notably, in the 96-well plate control, the initial measured concentration of tamoxifen from the 100 μM stock at 0 h was 36.6 μM, indicating a recovery rate of 36.6%. We attribute the loss of the compound to tamoxifen’s high binding affinity to albumin present in fetal bovine serum (FBS) in cell culture media.^56,57^ When 100 μM tamoxifen was applied to the cells cultured in the coated and uncoated MPS devices as well as in standard 96-well plates, distinct differences in cell viability were observed across the three conditions. In the 96-well plates, tamoxifen exposure resulted in a complete loss of viability in both DECs and AECs. In the Parylene-C-coated MPS devices, DECs exhibited similarly low viability; by contrast, cells remained largely viable (78.0 ± 2.90%) in uncoated devices. AECs demonstrated a similar trend, highly cytotoxic in both 96-well plates and coated MPS, but substantially higher viability was observed in uncoated devices (34.9 ± 2.42%) (Figure 5A). It is important to note that while uncoated devices showed higher viability than coated devices, tamoxifen still induced large amounts of cell death under both device conditions. These results show the effect of the Parylene-C coating in assessing the bioavailability and cytotoxic effects of lipophilic drugs in PDMS-based MPS devices.

**Figure 5:**
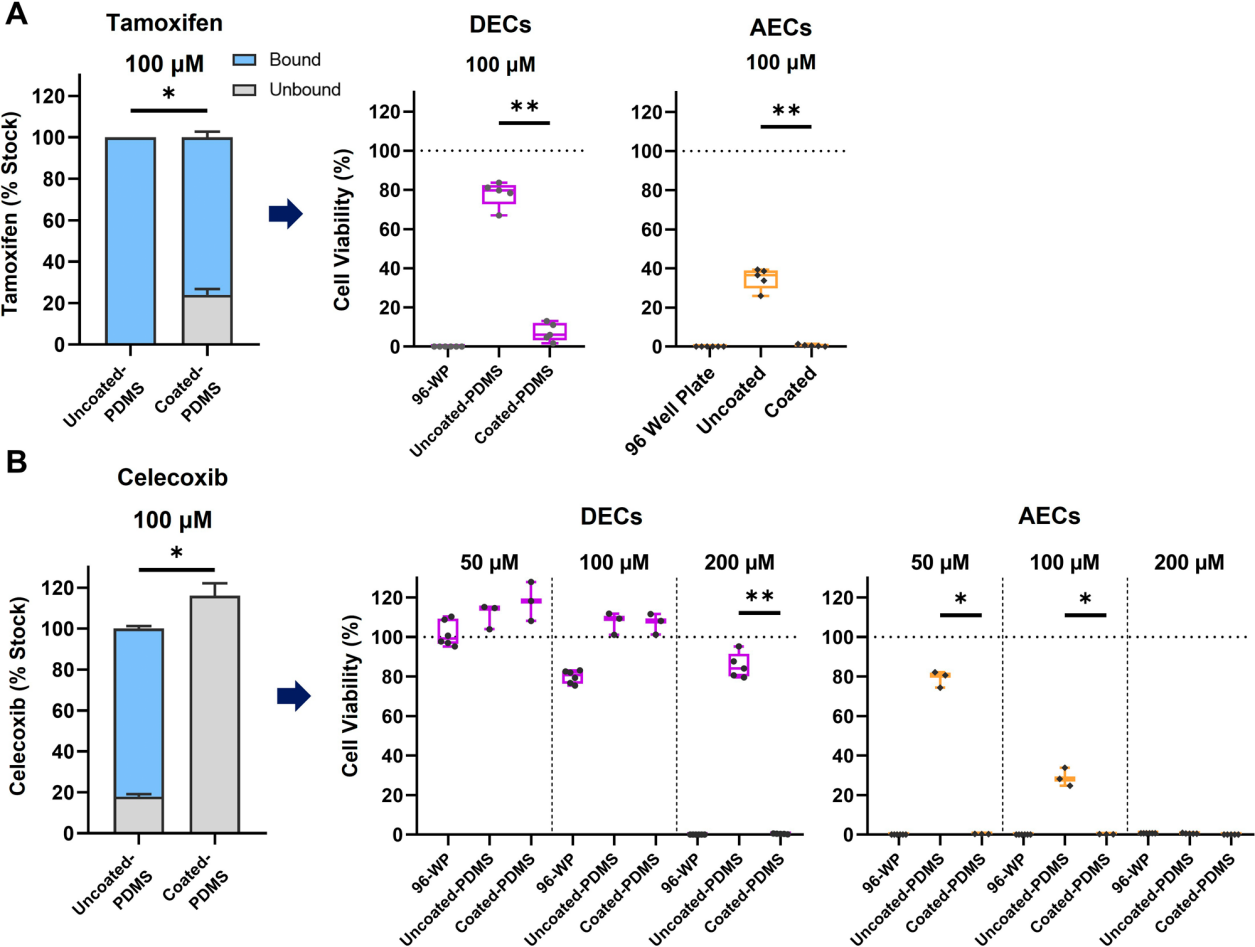
Assessment of tamoxifen (A) and celecoxib (B) bioavailability in coated vs uncoated PDMS-based MPS devices and its impact on the viability of DECs and AECs. Cells were exposed to 100 μM tamoxifen (A) and 50, 100, and 200 μM celecoxib (B) for 48 h in three different culture formats: standard 96-well plates, uncoated PDMS devices, and coated PDMS devices, to evaluate how surface coating influences drug adsorption and cellular response. Values for drug adsorption were normalized to the initial concentration at 0 h and are presented as stacked bars of % stock (mean ± SEM), and statistical analysis (unbound drug concentration [gray bars] in coated versus uncoated devices) was conducted using the Mann–Whitney U test and Student’s *t*-test, with statistical significance indicated as *p < 0.05 (*n* = 3). Values for cellular response are presented as a boxplot (median, interquartile range, max–min), and statistical analysis (coated vs uncoated devices) was conducted using the Mann–Whitney U test and Student’s *t*-test, with statistical significance indicated as *p < 0.05 and **p < 0.01 (*n* = 3–6).

Similarly, when celecoxib was introduced into the devices, only 18.0 ± 1.19% of the stock (measured concentration: 17.3 ± 1.61 μM) was recovered from uncoated devices, compared to 116 ± 6.08% (measured concentration: 111.5 ± 8.26 μM) in the coated devices (Figure 5B), indicating a significant reduction in celecoxib adsorption with the Parylene-C coating. The initial measured concentration of celecoxib from the 100 μM stock at 0 h was 96.1 μM in the 96-well plate control. To assess cellular responses to celecoxib in the MPS devices, three concentrations (50, 100, and 200 μM) were tested. In 96-well plate cultures, celecoxib exposure led to a complete loss of viability in AECs at all concentrations. However, DECs retained higher viability at lower concentrations, 102 ± 2.62% at 50 μM and 79.9 ± 1.33% at 100 μM, but then reached 0% at 200 μM. In the MPS experiments, DECs exhibited comparable viability between coated (118 ± 5.70% and 107 ± 3.08%) and uncoated (111 ± 3.66% and 107 ± 1.33%) conditions at 50 and 100 μM exposures. However, at 200 μM, DECs showed near-zero viability in Parylene-C-coated devices (0.41 ± 0.06%), similar to that seen in the 96-well-plate experiment, while maintaining significantly higher viability in uncoated devices (85.5 ± 2.84%). AECs demonstrated a similar trend at 50 and 100 μM, with minimal viability in coated devices (0.31 ± 0.02% and 0.20 ± 0.03%, respectively) and significantly higher viability in uncoated devices (79.1 ± 2.41% and 28.9 ± 2.64%). At 200 μM, however, AECs exhibited complete loss of viability in both coated and uncoated conditions (Figure 5B). These results again clearly show that lipophilic drugs can induce cytotoxicity in coated and uncoated devices. However, the bioavailability is greater in coated devices, highlighting the importance of the Parylene-C coating in assessing the effects of drugs in PDMS-based MPS devices.

In contrast, small-molecule drugs such as aspirin (coated: 25.4 ± 2.31% of stock, measured: 9.4 ± 1.21 μM; uncoated: 25.7 ± 1.36% of stock, measured: 10.2 ± 0.26 μM; initial measured concentration of 100 μM stock at 0 h: 37.0 μM) and sofosbuvir (coated: 103 ± 3.24% of stock, measured: 104.1 ± 4.61 μM; uncoated: 106 ± 4.50% of stock, measured: 106.8 ± 6.40 μM; initial measured concentration of 100 μM stock at 0 h: 100.7 μM) exhibited negligible binding differences between the coated and uncoated devices (Figure 6A,B). However, aspirin also appears to demonstrate a binding interaction with proteins present in the cell culture medium, as reported previously,^58–60^ and thus expected.

**Figure 6:**
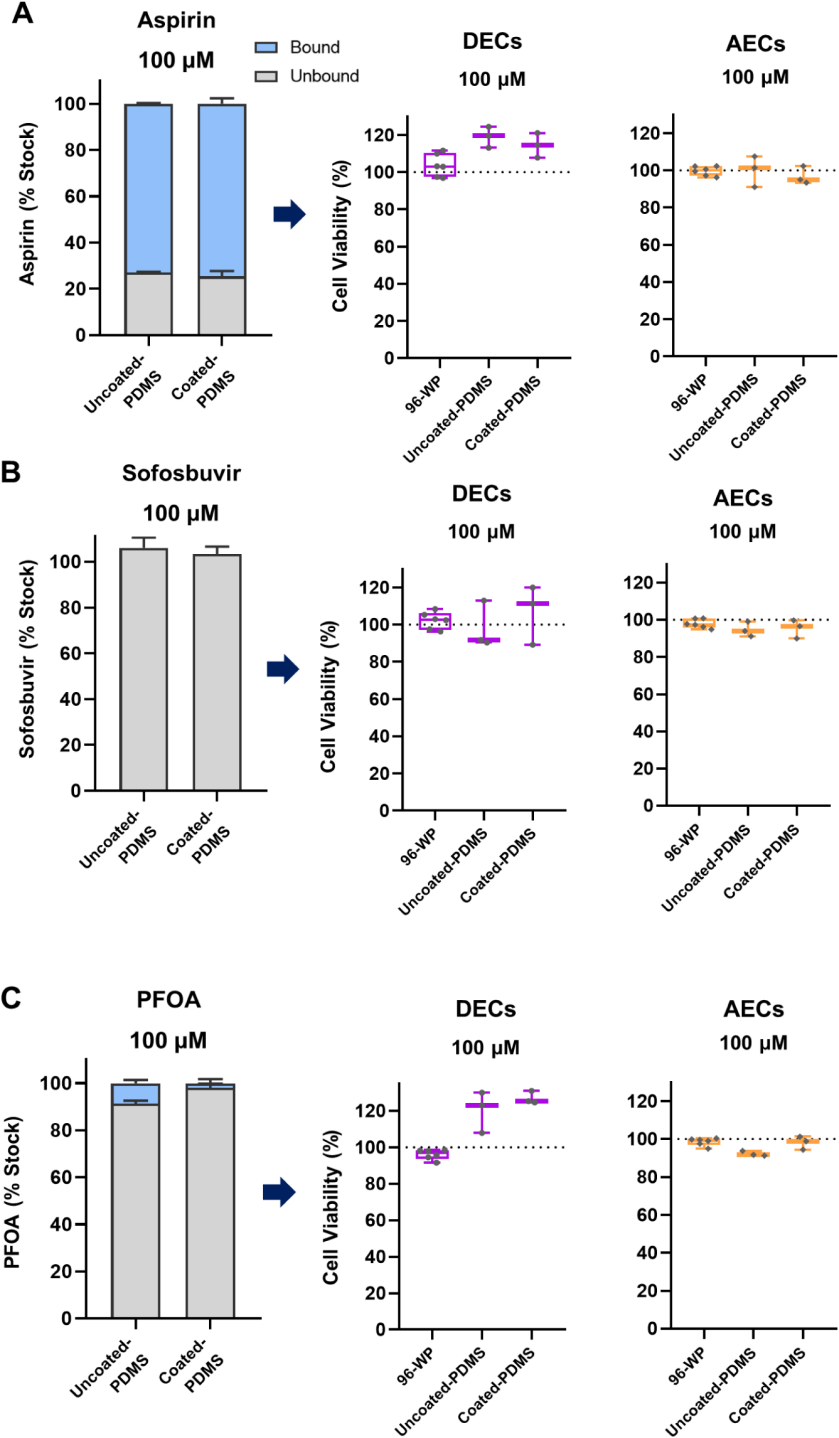
Evaluation of the bioavailability of (A) aspirin, (B) sofosbuvir, and (C) PFOA in coated vs uncoated PDMS-based MPS devices and their effects on DEC and AEC cell viability. Cells were exposed to 100 μM aspirin (A), sofosbuvir (B), and PFOA (C) for 48 h in three different culture formats: standard 96-well plates, uncoated PDMS devices, and coated PDMS devices, to evaluate how surface coating influences compound adsorption and cellular response. Values for compound adsorption were normalized to the initial concentration at 0 h and are presented as stacked bars of % stock (mean ± SEM), and statistical analysis (unbound compound concentration [gray bars] in coated versus uncoated) was conducted using the Mann–Whitney U test and Student’s *t*-test, where no significant differences were observed (*n* = 2–3). Values for cellular response are presented as a boxplot (median, interquartile range, max–min), and statistical analysis (coated vs uncoated devices) was conducted using the Mann–Whitney U test and Student’s *t*-test, where no significant differences were observed (*n* = 3–6).

Finally, we have tested the environmental pollutant PFOA, as there has been continuing interest in better understanding the potential toxic effects of PFAS-family chemicals.^61,62^ Here, PFOA (initial measured concentration of 100 μM stock: 109.1 μM) showed no significant adsorption differences between coated (98.1 ± 1.74% of stock, measured: 106.9 ± 2.68 μM) and uncoated devices (91.2 ± 1.34% of stock, measured: 99.5 ± 2.06 μM) (Figure 6C).

## 4 Discussion

Parylene-C is a biocompatible polymer that is well-established and extensively used for coating medical devices and implants due to its ability to form uniform, conformal, pinhole-free, robust air and liquid barriers.^40,41^ These properties make it an ideal choice for applications requiring long-term durability and interaction with biological systems.^37,63^ Parylene-C has demonstrated an excellent ability to prevent the adsorption of lipophilic small molecules, thereby enhancing the functionality and reliability of various biomedical devices.^41^ Its utility extends to improving experimental consistency and device performance in biological studies, including chemical screening.

The coating process is straightforward and less labor-intensive compared to other coating methods because it can be conducted at room temperature. Additionally, the coating process is solvent-free, eliminating the risks associated with chemical residues, which is particularly beneficial for maintaining cell growth and viability in biological applications. However, the excellent barrier properties of Parylene-C render coated devices essentially airtight,^48,64^ which can potentially restrict gas exchange to cells cultured within PDMS-based systems and raise concerns about reduced viability or impaired function. To mitigate these limitations, our MPS device was intentionally designed with large inlet and chamber reservoirs (5 mm diameter) to increase the media–air interface and maintain adequate oxygenation under static culture conditions. This design supports oxygen transfer comparable to that achieved in standard cell culture plastics (e.g., 96-well plates), which were included in this study as controls. Consistent with this, cell viability in the coated devices remained unaffected in the presence of the Parylene-C coating.

While Parylene-C does lower the intrinsic gas permeability of PDMS, many MPS applications employing shallow channels and short-term cultures (<3–5 days) are not typically limited by oxygen diffusion, even with reduced permeability. Importantly, prior studies have directly measured how oxygen permeability is impacted by Parylene-C coating,^48,64,65^ where applications requiring deeper channels, higher metabolic activity, or extended culture durations may necessitate active perfusion to avoid hypoxic conditions. Overall, our experimental findings confirm that Parylene-C coating is biocompatible and nontoxic under the conditions relevant to this study, while offering substantial advantages for reducing small-molecule adsorption (Figure 3, Figure S2, and Figure S3).

Our experimental findings further suggest that the small-molecule anti-inflammatory drug celecoxib exhibits significant adsorption to uncoated PDMS surfaces, resulting in elevated cellular viability in uncoated devices compared with both Parylene-C-coated devices and 96-well plate cultures (Figure 5B). This suggests that surface adsorption reduces celecoxib’s effective bioavailability, thereby attenuating its cytotoxic effects. A comparable pattern was observed with tamoxifen, a drug known to either adsorb to PDMS surfaces or bind to serum albumin in cell culture media. Tamoxifen is highly lipophilic and binds extensively to serum albumin, thereby reducing the free, bioavailable fraction available to interact with cells. This protein-binding effect can lead to lower apparent toxicity at a given nominal concentration, since only the unbound drug can cross cell membranes and exert its biological effect.^56,57^ While considerations of free vs bound drug concentrations in cell-based models are important for *in vitro*-to-*in vivo* extrapolations, adjustments for free concentrations do not necessarily enhance classification or *in vitro/in vivo* concordance.^66^ However, in both 96-well plates and coated PDMS devices, tamoxifen reduced the viability of DECs and AECs, while uncoated devices showed considerably higher viability, again indicating reduced drug exposure due to adsorption, even after binding to serum albumin (Figure 5A). Nevertheless, it is worth noting that even uncoated devices treated with celecoxib and tamoxifen would have induced significant cell death compared to untreated controls and would not pass the 20% cell death threshold for “good viability” commonly used in the reproductive MPS field. This observation further supports the hypothesis that drug adsorption to PDMS reduces the bioavailable concentration of the drug to the cells, leading to artificially elevated cellular viability in uncoated devices. Here, the surface modification of the PDMS device with Parylene-C mitigated this effect by preserving drug availability, thereby enabling more accurate assessment of drug toxicity. Consistent with this functional improvement, the Parylene-C-coated PDMS device maintained a consistently lower contact angle relative to uncoated PDMS, indicating that the coating remained hydrophilic and did not undergo degradation during incubation. This stable coating correlates with the observed reduction in adsorption of highly lipophilic molecules such as tamoxifen and celecoxib.

Moreover, small-molecule drugs such as aspirin and sofosbuvir demonstrated negligible differences in cell viability between a 96-well plate, coated, and uncoated devices for both cell types tested (Figure 6A,B). Similarly, exposure to the environmental toxicant PFOA revealed no significant differences in cell viability of both cell types across 96-well plates and coated and uncoated devices (Figure 6C). These findings indicate that Parylene-C coating may not be necessary for all molecular testing in PDMS-based MPS devices, as adsorption to PDMS varies significantly depending on the compound’s physicochemical properties. While lipophilic compounds with higher logP generally exhibit greater adsorption, PFOA is an outlier, as its amphiphilic nature, with a hydrophobic fluorinated tail and ionizable hydrophilic carboxylic acid head, leads to atypical adsorption behavior (Table S1). However, for PDMS-based MPS devices to be used for a broad range of molecular testing, performing Parylene-C coating, as there is no negative impact on cell behaviors and the coating process is relatively easy, low-cost, and high-throughput, becomes an attractive option.

Parylene-C coating on PDMS-based cell culture devices has been previously explored, particularly for its ability to reduce the adsorption of certain drugs^52^ and fluorescent dyes.^67^ Despite these studies, this surface modification technique has not been widely adopted in the MPS field, where drug discovery and toxicity testing using MPS are increasingly prevalent. Beyond the compounds investigated in this study, the Parylene-C coating strategy has broader applicability across MPS, allowing for the evaluation of substances with a wide range of physicochemical properties. MPS is being used in toxicology and pharmacokinetic studies of highly lipophilic substances. Additionally, numerous lipophilic drugs have also shown strong binding to or absorption into PDMS, thus complicating the interpretation of the results from studies using MPS.^18,68–70^ In these contexts, adsorption-related underdosing can considerably affect cellular responses, mask toxicity signals, and reduce the reproducibility of MPS-based mechanistic studies. By mitigating this challenge, Parylene-C coating can enhance the accuracy of dosing characterization and *in vitro-*to-*in vivo* extrapolation and improve the reliability and translational relevance of PDMS-based MPS platforms across a wide range of pharmacological and toxicological applications.

While this study primarily focused on establishing Parylene-C coating as a practical strategy to reduce small-molecule adsorption in PDMS-based MPS devices, the improved compound recovery demonstrated here lays the foundation for the design of future mechanistic toxicological studies. The enhanced bioavailability afforded by the coating, verified using a set of compounds with different physicochemical properties, will allow future studies with other substances to provide reliable and reproducible mechanistic insights into cellular responses to a broad range of compounds.

In addition, the study demonstrates that surface modification of PDMS with Parylene-C does not adversely affect the transport or propagation of molecules between microchannel-interconnected chambers, indicating that device functionality remains intact. These findings collectively highlight the critical importance of considering material-drug interactions when designing and utilizing PDMS-based MPS for biomedical and pharmaceutical applications. Although we did not vary coating thickness, future studies may investigate how thinner coatings (e.g., 0.5–1 μm) or alternative parylene chemistries influence adsorption reduction and transport properties. By mitigating adsorption while maintaining molecular transport, the Parylene-C coating can enhance dose accuracy and improve the reliability and translational relevance of PDMS-based MPS platforms across a wide range of pharmacological and toxicological applications.

## 5 Conclusion

To ensure reliable drug and chemical testing using MPS models, evaluating the potential adsorption or absorption of these substances to device surfaces is crucial. Considerable drug/molecule adsorption can diminish the bioavailability, leading to inaccurate efficacy and cytotoxicity testing results. In this study, we demonstrated that Parylene-C coating on PDMS-based MPS devices can function as an effective coating to minimize lipophilic drug/molecule adsorption, which was demonstrated through molecular propagation kinetic study, cytotoxicity assessment, and mass spectroscopic analysis of available drugs/molecules postcultivation. By mitigating lipophilic small molecule drug adsorption, the Parylene-C coating enhances bioavailability, ensuring more accurate and dependable outcomes in drug/chemical cytotoxicity and efficacy assessments. The relatively easy and fully automated coating process, where over 50 devices can be easily coated in a single processing step, makes this method well-suited for preparing many MPS devices in parallel and thus is expected to have broad applicability in the MPS field.

## Supplementary Material



## Data Availability

The data that support the findings of this study are available upon request from the corresponding authors.

## Notes

Supplementary experiments provide supporting evidence for device fabrication, Parylene-C coating integrity, long-term cell culture viability, disease model viability, dose–response analyses of tamoxifen and celecoxib in 96-well plate assays, and characterization of the physicochemical properties of the model compounds (PDF)

## References

[1] Hughes J. P., Rees S., Kalindjian S. B., Philpott K. L. (2011). Principles of early
drug discovery. Br. J. Pharmacol..

[2] Sertkaya A., Beleche T., Jessup A., Sommers B. D. (2024). Costs of Drug Development
and Research and Development Intensity in the US, 2000–2018. JAMA Netw. Open.

[3] Richardson L. S., Kammala A. K., Kim S., Lam P. Y., Truong N., Radnaa E., Urrabaz-Garza R., Han A., Menon R. (2023). Development
of oxidative stress-associated disease models using feto-maternal
interface organ-on-a-chip. FASEB J..

[4] Ingber D. E. (2022). Human organs-on-chips
for disease modelling, drug development and personalized medicine. Nat. Rev. Genet..

[5] Richardson L., Jeong S., Kim S., Hart A., Menon R. (2019). Amnion membrane
organ-on-chip: an innovative approach to study cellular interactions. FASEB J..

[6] Kim S., Richardson L., Radnaa E., Chen Z., Rusyn I., Menon R., Han A. (2022). Molecular mechanisms of environmental
toxin cadmium at the feto-maternal interface investigated using an
organ-on-chip (FMi-OOC) model. J. Hazard Mater..

[7] Lam P. Y., Kim S., Jung H., Cherukuri R., Menon R., Han A. (2025). Enhanced operation
of female reproductive microphysiological system (MPS) for rapid mechanistic
study. Micro Nano Syst. Lett..

[8] Vidal M. S., Richardson L. S., Kumar Kammala A., Kim S., Lam P. Y., Cherukuri R., Thomas T. J., Bettayeb M., Han A., Rusyn I., Menon R. (2024). Endocrine-disrupting compounds and
their impact on human placental function: evidence from placenta organ-on-chip
studies. Lab Chip.

[9] Cherukuri R., Kammala A. K., Thomas T. J., Saylor L., Richardson L., Kim S., Ferrer M., Acedo C., Song M. J., Gaharwar A. K., Menon R., Han A. (2024). High-Throughput 3D-Printed Model
of the Feto-Maternal Interface for the Discovery and Development of
Preterm Birth Therapies. ACS Appl. Mater. Interfaces.

[10] Saylor L. M., Cherukuri R., Kammala A. K., Richardson L., Ferrer M., Antich C., Frebert S., Han A., Menon R. (2025). Exosomal Delivery of Interleukin-10 Reduces Infection-Associated
Inflammation in a 3D-Printed Model of a Humanized Feto-Maternal Interface. FASEB J..

[11] Dehne E. M., Hasenberg T., Marx U. (2017). The ascendance of microphysiological
systems to solve the drug testing dilemma. Future
Sci. OA.

[12] Wang K., Man K., Liu J., Liu Y., Chen Q., Zhou Y., Yang Y. (2020). Microphysiological Systems: Design, Fabrication, and Applications. ACS Biomater. Sci. Eng..

[13] Mou L., Mandal K., Mecwan M. M., Hernandez A. L., Maity S., Sharma S., Herculano R. D., Kawakita S., Jucaud V., Dokmeci M. R., Khademhosseini A. (2022). Integrated
biosensors for monitoring microphysiological systems. Lab Chip.

[14] Zhang Y. S., Aleman J., Shin S. R., Kilic T., Kim D., Mousavi Shaegh S. A., Massa S., Riahi R., Chae S., Hu N., Avci H., Zhang W., Silvestri A., Sanati Nezhad A., Manbohi A., De Ferrari F., Polini A., Calzone G., Shaikh N., Alerasool P., Budina E., Kang J., Bhise N., Ribas J., Pourmand A., Skardal A., Shupe T., Bishop C. E., Dokmeci M. R., Atala A., Khademhosseini A. (2017). Multisensor-integrated
organs-on-chips platform for automated and continual in situ monitoring
of organoid behaviors. Proc. Natl. Acad. Sci.
U. S. A..

[15] Waheed S., Cabot J. M., Macdonald N. P., Kalsoom U., Farajikhah S., Innis P. C., Nesterenko P. N., Lewis T. W., Breadmore M. C., Paull B. (2017). Enhanced physicochemical
properties of polydimethylsiloxane based
microfluidic devices and thin films by incorporating synthetic micro-diamond. Sci. Rep..

[16] Gokaltun A., Kang Y. B. A., Yarmush M. L., Usta O. B., Asatekin A. (2019). Simple Surface
Modification of Poly­(dimethylsiloxane) via Surface Segregating Smart
Polymers for Biomicrofluidics. Sci. Rep..

[17] Bakute N., Andriukonis E., Kasperaviciute K., Dobilas J., Sapurov M., Mozolevskis G., Stirke A. (2024). Microphysiological system with integrated
sensors to study the effect of pulsed electric field. Sci. Rep..

[18] Auner A. W., Tasneem K. M., Markov D. A., McCawley L. J., Hutson M. S. (2019). Chemical-PDMS
binding kinetics and implications for bioavailability in microfluidic
devices. Lab Chip.

[19] Toepke M. W., Beebe D. J. (2006). PDMS absorption
of small molecules and consequences
in microfluidic applications. Lab Chip.

[20] Wang J. D., Douville N. J., Takayama S., ElSayed M. (2012). Quantitative analysis
of molecular absorption into PDMS microfluidic channels. Ann. Biomed. Eng..

[21] Rodrigues P. M., Xavier M., Calero V., Pastrana L., Goncalves C. (2022). Partitioning
of Small Hydrophobic Molecules into Polydimethylsiloxane in Microfluidic
Analytical Devices. Micromachines (Basel).

[22] Sharma D., Lim R. Y. H., Pfohl T., Ekinci Y. (2021). Surface-modified elastomeric
nanofluidic devices for single nanoparticle trapping. Microsyst. Nanoeng..

[23] Zhou J., Khodakov D. A., Ellis A. V., Voelcker N. H. (2012). Surface modification
for PDMS-based microfluidic devices. Electrophoresis.

[24] Trantidou T., Elani Y., Parsons E., Ces O. (2017). Hydrophilic surface
modification of PDMS for droplet microfluidics using a simple, quick,
and robust method via PVA deposition. Microsyst.
Nanoeng..

[25] Sui G., Wang J., Lee C.-C., Lu W., Lee S. P., Leyton J. V., Wu A. M., Tseng H.-R. (2006). Solution-Phase
Surface
Modification in Intact Poly­(dimethylsiloxane) Microfluidic Channels. Anal. Chem..

[26] Wong I., Ho C.-M. (2009). Surface molecular
property modifications for poly­(dimethylsiloxane)
(PDMS) based microfluidic devices. Microfluid.
Nanofluid..

[27] Mader M., Rein C., Konrat E., Meermeyer S. L., Lee-Thedieck C., Kotz-Helmer F., Rapp B. E. (2021). Fused Deposition
Modeling of Microfluidic Chips in Transparent Polystyrene. Micromachines (Basel).

[28] Wen X., Yoshimoto K., Yamanaka M., Terada S., Kamei K.-I. (2021). In vitro
nonalcoholic fatty liver disease model with cyclo-olefin-polymer-based
microphysiological systems. Organs-On-A-Chip.

[29] Yamanaka M., Wen X., Imamura S., Sakai R., Terada S., Kamei K. I. (2021). Cyclo olefin
polymer-based solvent-free mass-productive microphysiological systems. Biomed. Mater..

[30] Nguyen O. T. P., Misun P. M., Hierlemann A., Lohasz C. (2024). A Versatile Intestine-on-Chip
System for Deciphering the Immunopathogenesis of Inflammatory Bowel
Disease. Adv. Healthcare Mater..

[31] Rimsa R., Galvanovskis A., Plume J., Rumnieks F., Grindulis K., Paidere G., Erentraute S., Mozolevskis G., Abols A. (2021). Lung on a Chip Development from Off-Stoichiometry Thiol-Ene Polymer. Micromachines (Basel).

[32] Shakeri A., Khan S., Jarad N. A., Didar T. F. (2022). The Fabrication
and Bonding of Thermoplastic Microfluidics: A Review. Materials (Basel).

[33] Giri K., Tsao C. W. (2022). Recent Advances in Thermoplastic Microfluidic Bonding. Micromachines (Basel).

[34] Chung C.-H., Kuo W.-C. (2024). Anti-corrosion application of parylene C film for stainless
steel fasteners in electroplating industry. AIP Adv..

[35] Buchwalder S., Borzi A., Diaz Leon J. J., Bourgeois F., Nicolier C., Nicolay S., Neels A., Zywitzki O., Hogg A., Burger J. (2022). Thermal Analysis of
Parylene Thin
Films for Barrier Layer Applications. Polymers
(Basel).

[36] Ortigoza-Diaz J., Scholten K., Larson C., Cobo A., Hudson T., Yoo J., Baldwin A., Weltman Hirschberg A., Meng E. (2018). Techniques and Considerations
in the Microfabrication of Parylene C Microelectromechanical Systems. Micromachines (Basel).

[37] Coelho B. J., Pinto J. V., Martins J., Rovisco A., Barquinha P., Fortunato E., Baptista P. V., Martins R., Igreja R. (2023). Parylene C
as a Multipurpose Material for Electronics and Microfluidics. Polymers (Basel).

[38] Yuvaraja S., Faber H., Kumar M., Xiao N., Maciel García G. I., Tang X., Anthopoulos T. D., Li X. (2024). Three-dimensional integrated
metal-oxide transistors. Nat. Electron..

[39] Stucchi E., Dell’erba G., Colpani P., Kim Y.-H., Caironi M. (2018). Low-Voltage
Printed, All-Polymer Integrated Circuits Employing a Low-Leakage and
High-Yield Polymer Dielectric. Adv. Electron.
Mater..

[40] Hao D., Lin J., Liu R., Pivetti C., Yamashiro K., Schutzman L. M., Sageshima J., Kwong M., Bahatyrevich N., Farmer D. L., Humphries M. D., Lam K. S., Panitch A., Wang A. (2023). A bio-instructive parylene-based conformal coating suppresses thrombosis
and intimal hyperplasia of implantable vascular devices. Bioact Mater..

[41] Golda-Cepa M., Engvall K., Hakkarainen M., Kotarba A. (2020). Recent progress on
parylene C polymer for biomedical applications: A review. Prog. Org. Coat..

[42] French P., Krijnen G., Roozeboom F. (2016). Precision in harsh environments. Microsyst. Nanoeng..

[43] Kim H., Lee J., Kim B., Byun H. R., Kim S. H., Oh H. M., Baik S., Jeong M. S. (2019). Enhanced Stability of MAPbI(3) Perovskite
Solar Cells using Poly­(p-chloro-xylylene) Encapsulation. Sci. Rep..

[44] Raos B. J., Simpson M. C., Doyle C. S., Graham E. S., Unsworth C. P. (2019). Evaluation
of parylene derivatives for use as biomaterials for human astrocyte
cell patterning. PLoS One.

[45] Humphrey, B. Using Parylene for Medical Substrate Coating; MDDI Qmed, 1996.

[46] SCS Different Types of Parylene; Specialty Coating Systems, 2023.

[47] Comparing Parylene Types: a Guide to Choosing the Right Variant for Your Application; Advanced Coating, 2024.

[48] Oppegard S.
C., Blake A. J., Williams J. C., Eddington D. T. (2010). Precise
control over the oxygen conditions within the Boyden chamber using
a microfabricated insert. Lab Chip.

[49] Mehta G., Mehta K., Sud D., Song J. W., Bersano-Begey T., Futai N., Heo Y. S., Mycek M. A., Linderman J. J., Takayama S. (2007). Quantitative measurement
and control of oxygen levels
in microfluidic poly­(dimethylsiloxane) bioreactors during cell culture. Biomed. Microdev..

[50] Ranjit
Prakash A., Adamia S., Sieben V., Pilarski P., Pilarski L. M., Backhouse C. J. (2006). Small volume PCR in PDMS biochips
with integrated fluid control and vapour barrier. Sens. Actuators, B.

[51] Heo Y. S., Cabrera L. M., Song J. W., Futai N., Tung Y. C., Smith G. D., Takayama S. (2007). Characterization and
resolution of
evaporation-mediated osmolality shifts that constrain microfluidic
cell culture in poly­(dimethylsiloxane) devices. Anal. Chem..

[52] Wang L., Yu L., Grist S., Cheung K. C., Chen D. D. Y. (2017). Different in
vitro cellular responses to tamoxifen treatment in polydimethylsiloxane-based
devices compared to normal cell culture. J.
Chromatogr. B: Anal. Technol. Biomed. Life Sci..

[53] Richardson L., Radnaa E., Lintao R. C. V., Urrabaz-Garza R., Maredia R., Han A., Sun J., Menon R. (2023). A Microphysiological
Device to Model the Choriodecidual Interface Immune Status during
Pregnancy. J. Immunol..

[54] Menon R., Radnaa E., Behnia F., Urrabaz-Garza R. (2020). Isolation
and characterization human chorion membrane trophoblast and mesenchymal
cells. Placenta.

[55] Radnaa E., Urrabaz-Garza R., Elrod N. D., de Castro Silva M., Pyles R., Han A., Menon R. (2022). Generation and characterization
of human Fetal membrane and Decidual cell lines for reproductive biology
experiments†. Biol. Reprod..

[56] Bourassa P., Dubeau S., Maharvi G. M., Fauq A. H., Thomas T. J., Tajmir-Riahi H. A. (2011). Locating the binding sites of anticancer tamoxifen
and its metabolites 4-hydroxytamoxifen and endoxifen on bovine serum
albumin. Eur. J. Med. Chem..

[57] Kedjouar B., de Medina P., Oulad-Abdelghani M., Payre B., Silvente-Poirot S., Favre G., Faye J. C., Poirot M. (2004). Molecular characterization
of the microsomal tamoxifen binding site. J.
Biol. Chem..

[58] Aarons L., Clifton P., Fleming G., Rowland M. (1980). Aspirin binding and
the effect of albumin on spontaneous and enzyme-catalysed hydrolysis. J. Pharm. Pharmacol..

[59] Lee S., Johnson D., Klein J., Eppler J. (1995). Protein binding of
acetylsalicylic acid and salicylic acid in porcine and human serum. Vet. Hum. Toxicol..

[60] Nafisi S., Bagheri Sadeghi G., PanahYab A. (2011). Interaction of aspirin and vitamin
C with bovine serum albumin. J. Photochem. Photobiol.
B.

[61] Tian H., Gaines C., Launi L., Pomales A., Vazquez G., Goharian A., Goodnight B., Haney E., Reh C. M., Rogers R. D. (2022). Understanding Public
Perceptions of Per- and Polyfluoroalkyl
Substances: Infodemiology Study of Social Media. J. Med. Internet Res..

[62] Solan M. E., Park J. A. (2024). Per- and poly-fluoroalkyl
substances (PFAS) effects
on lung health: a perspective on the current literature and future
recommendations. Front. Toxicol..

[63] Lin C. Y., Lou W. S., Chen J. C., Weng K. Y., Shih M. C., Hung Y. W., Chen Z. Y., Wang M. C. (2020). Bio-Compatibility
and Bio-Insulation of Implantable Electrode Prosthesis Ameliorated
by A-174 Silane Primed Parylene-C Deposited Embedment. Micromachines (Basel).

[64] Rivera K. R., Yokus M. A., Erb P. D., Pozdin V. A., Daniele M. (2019). Measuring
and regulating oxygen levels in microphysiological systems: design,
material, and sensor considerations. Analyst.

[65] Yoon J. Y., Ahn Y., Schröder U. (2018). Parylene C-coated
PDMS-based microfluidic
microbial fuel cells with low oxygen permeability. J. Power Sources.

[66] Lin H.-C., Baltazar M. T., Cable S., Ford L. C., Middleton A. M., Nicol B., Punt A., Reynolds J., Rusyn I., Chiu W. A. (2025). Sensitivity analysis
of the inputs for bioactivity-exposure
ratio calculations in a NAM-based systemic safety toolbox. NAM J..

[67] Sasaki H., Onoe H., Osaki T., Kawano R., Takeuchi S. (2010). Parylene-coating
in PDMS microfluidic channels prevents the absorption of fluorescent
dyes. Sens. Actuators, B.

[68] Moyer H. L., Kim S., Lam B. P., Richardson L. S., Tsai H. D., Ford L. C., Lin H. C., Chiu W. A., Menon R., Han A., Rusyn I. (2025). Fetal response
to maternal exposures of environmental chemicals:
Utility of a four-cell human feto-maternal interface organ-on-chip. Chem. Biol. Interact..

[69] Grindulis K., Matusevica N. G., Kozlova V., Rimsa R., Klavins K., Mozolevskis G. (2025). Sorption and release of small molecules in PDMS and
COC for Organs on chip. Sci. Rep..

[70] Grant J., Özkan A., Oh C., Mahajan G., Prantil-Baun R., Ingber D. E. (2021). Simulating drug
concentrations in PDMS microfluidic
organ chips. Lab Chip.

