# Supplementary material for: Reducing Small Molecule Adsorption in a PDMS-Based Microphysiological System of the Female Reproductive Tract via Parylene‑C Coating to Improve Mechanistic Studies
Source: ACS Appl Mater Interfaces. 2026 Jan 6;18(2):3565–77. doi: 10.1021/acsami.5c20917 (PMC12828717; doi:10.1021/acsami.5c20917)
Supplement: Supplementary file 1 [file am5c20917_si_001.pdf]

Supporting Information

Reducing Small Molecule Adsorption in a PDMS-Based Microphysiological System of the Female Reproductive System via Parylene-C Coating to Improve Mechanistic Studies

Rahul Cherukuri[1†], Sungjin Kim[1†], Haley Moyer[2], Hayley Jesse[2], Po Yi Lam[1], Lauren Richardson[3], Ananth Kumar Kammala[3], Ramkumar Menon[3], Ivan Rusyn[2], and Arum Han[1,4,5*]

[1]Department of Electrical and Computer Engineering, Texas A&M University, College Station, Texas, 77840, USA.
[2]Department of Veterinary Physiology and Pharmacology, College of Veterinary Medicine and Biomedical Sciences, Texas A&M University, College Station, Texas, 77840, USA.
[3]Division of Basic Science and Translational Research, Department of Obstetrics & Gynecology, The University of Texas Medical Branch at Galveston, Galveston, Texas, 77555, USA.
[4]Department of Biomedical Engineering, Texas A&M University, College Station, Texas, 77840 USA.
[5]Department of Chemical Engineering, Texas A&M University, College Station, Texas, 77840, USA.

[†]Both authors contributed equally to this manuscript

*** Corresponding author:**
Arum Han, PhD
Professor, Department of Electrical and Computer Engineering
Department of Biomedical Engineering
Department of Chemical Engineering
Texas A&M University
E-mail: arum.han@ece.tamu.edu

[†]These authors contributed equally to this work

1. Methods

1.1. Long-term (7-day) cell culture

MPS devices (coated and uncoated) were sterilized with 70% ethanol for 15 min, washed three times with PBS, and subsequently rinsed three times with complete DMEM/F12 to prepare for cell seeding. DECs and AECs were then introduced into their respective chambers: 65,000 DECs in 160 µL of DEC-specific medium were seeded into the outer compartment, and 30,000 AECs in 65 µL of AEC-specific medium were seeded into the inner compartment. Devices were maintained at 37°C and 5% CO_2 for 7 days, with media replenished every 48 h. Cell viability was assessed on days 2, 4, and 7.

1.2. Surface Characterization

The surfaces of the PDMS-based MPS devices (coated and uncoated) were characterized for hydrophilicity/hydrophobicity, coating integrity (pinhole-free coverage), and resistance to degradation using contact angle quantitative measurements at 0 h, 1 day, 2 days, 4 days, and 7 days, and scanning electron microscopy (SEM) qualitative images. PDMS molds were pre-treated with oxygen plasma (Harrick Plasma, Ithaca, USA) for 120 s to enhance hydrophilicity for easier cell attachment and medium loading. All PDMS molds (plasma-treated and non-treated) were submerged in cell culture medium and incubated for the full duration of each time point. Before each characterization step, devices were washed with 70% ethanol followed by PBS to prevent contamination, and then dried using nitrogen gas. The contact angle measurements were conducted using a Theta Flex Optical Tensiometer (Biolin Scientific, Sweden), and the surface roughness was measured using a Dektak Pro Profilometer (Bruker, Billerica, MA) at each time point.

2. Figures and Tables

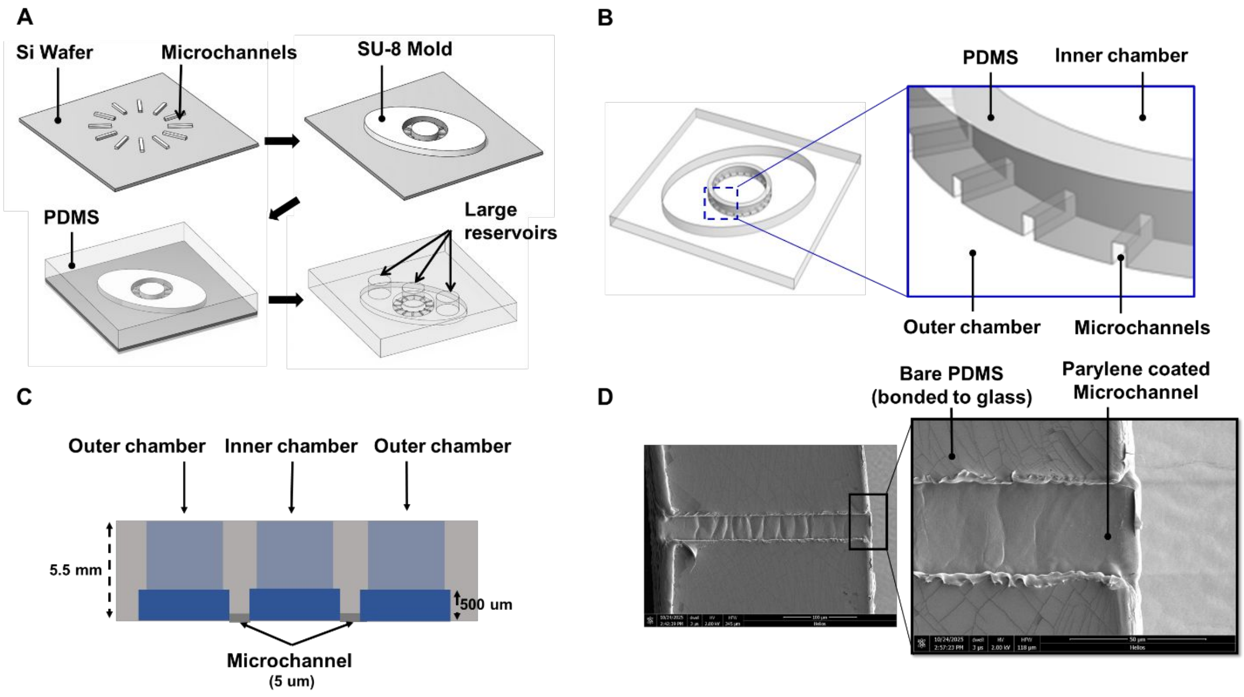

Figure S1. Design and fabrication of the PDMS-based two-chamber MPS device. **A.** Microfabrication and assembly steps for the MPS device. Two SU-8 layers with different thicknesses were patterned on top of a silicon substrate to form the microchannels and the 2 cell culture chambers. PDMS devices were replicated from the SU-8 master using a soft lithography process, and 5-mm diameter reservoirs were punched out, followed by bonding onto glass substrates. **B.** Schematic illustration of the PDMS-based two-chamber MPS device. **C.** Cross-sectional view of the PDMS-based two-chamber MPS device. **D.** Scanning electron microscopy (SEM) images of parylene-coated microchannel.

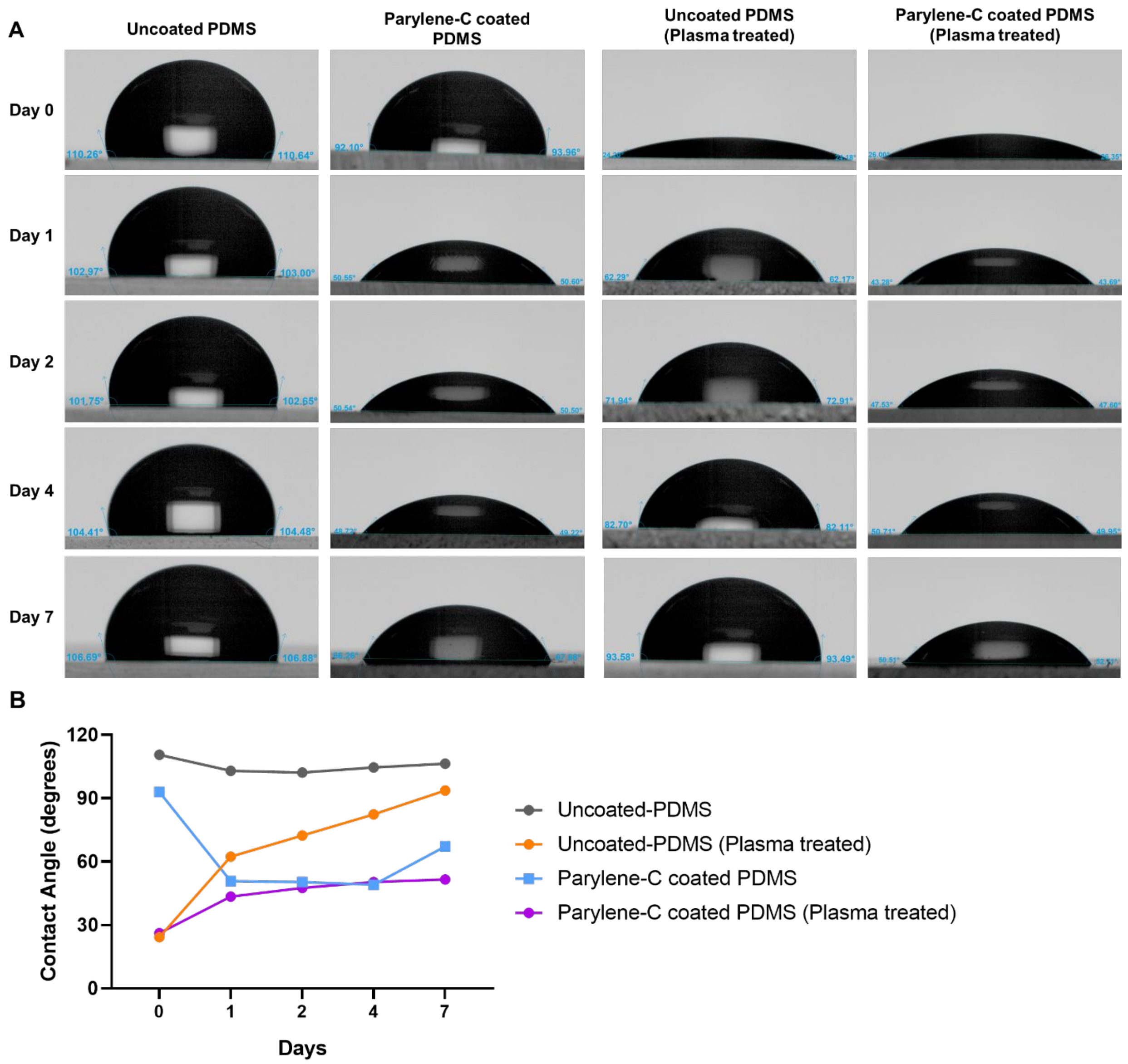

Figure S2. Comparison of contact angle measurements for coated and uncoated PDMS surfaces, with and without plasma treatment, demonstrates that Parylene-coated PDMS remains more hydrophilic than uncoated PDMS over extended incubation periods (7 days) in cell culture medium. Qualitative (**A**) and quantitative (**B**) assessments were performed on days 0, 1, 2, 4, and 7.

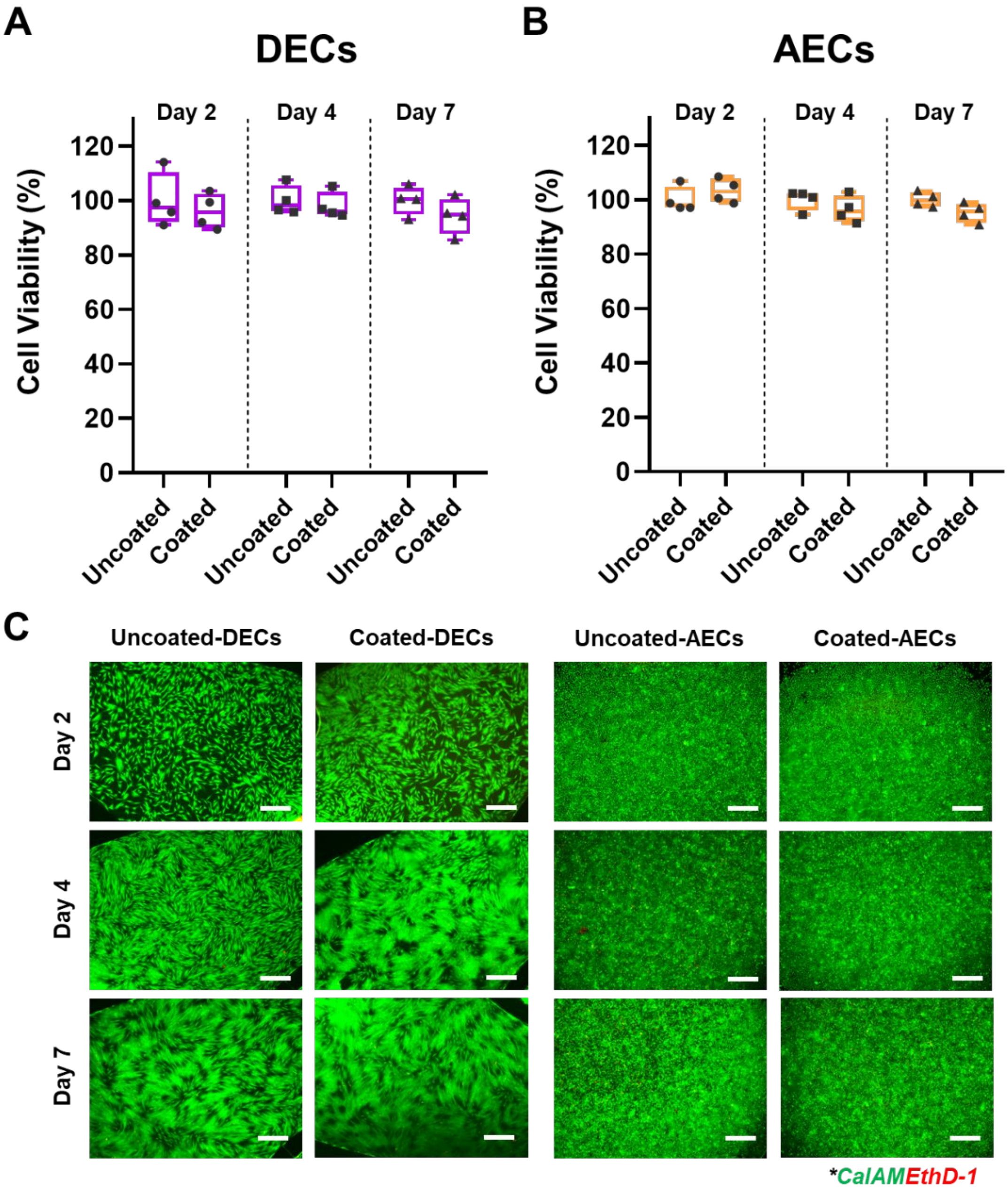

Figure S3. Cell viability of DECs and AECs cultured within coated and uncoated MPS devices for up to 7 days was evaluated using Alamar Blue assay (**A-B**) and Live Dead staining (calcein AM and ethidium homodimer) (**C**) Boxplots (median, interquartile range, max-min) are shown, and statistical analysis was performed using the Mann-Whitney U and Student's t-test, where no significant differences were observed at $p<0.05$ (n=5). Scale bar = 50 μm.

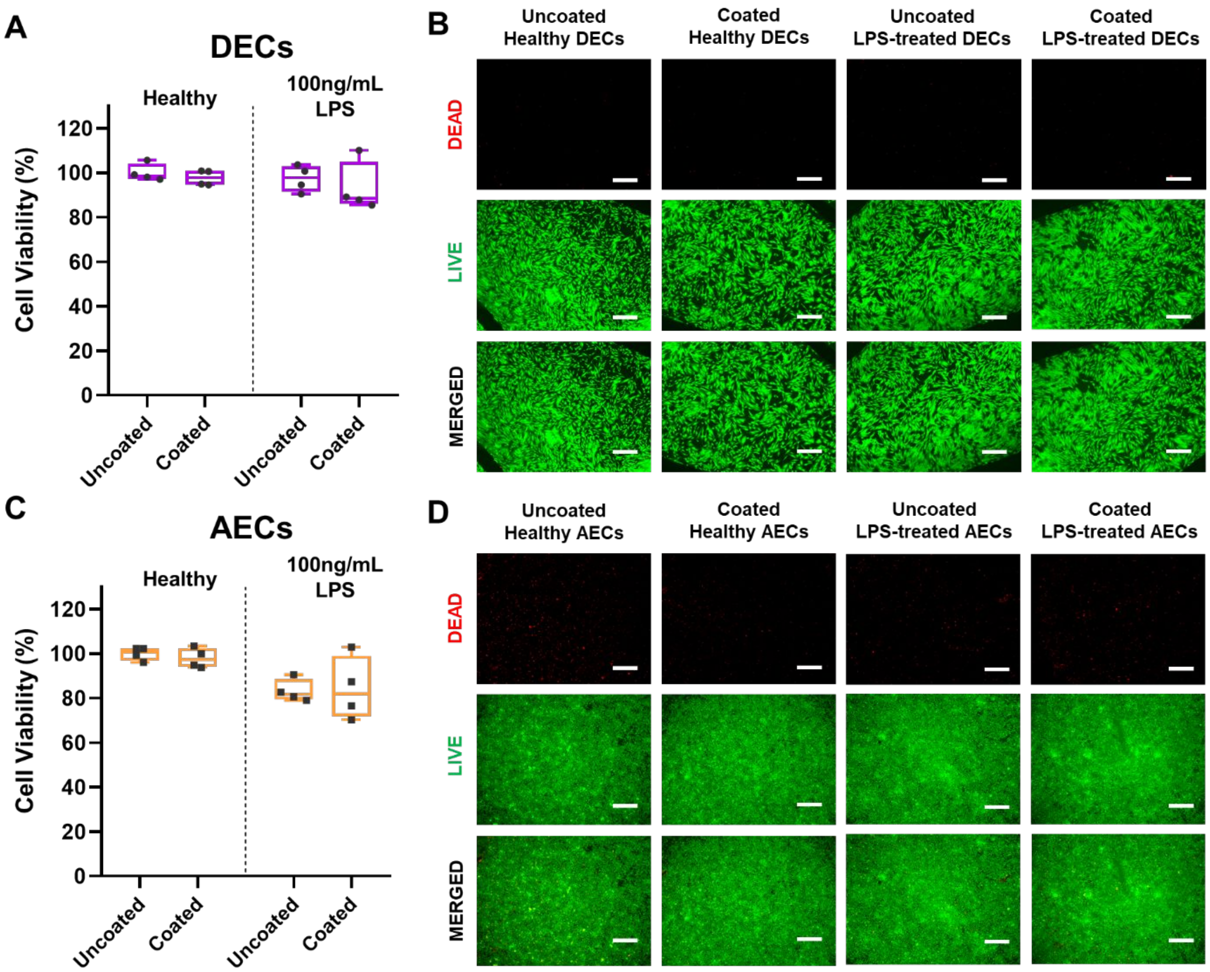

Figure S4. Cell viability of DECs (**A-B**) and AECs (**C-D**) cultured within coated and uncoated MPS devices exposed to 100 ng/mL LPS was evaluated using Alamar Blue assay and Live Dead staining. Boxplots (median, interquartile range, max-min) are shown, and statistical analysis was performed using the Mann-Whitney U and Student's t-test, where no significant differences were observed at $p<0.05$ (n=5). Scale bar = 50 μm.

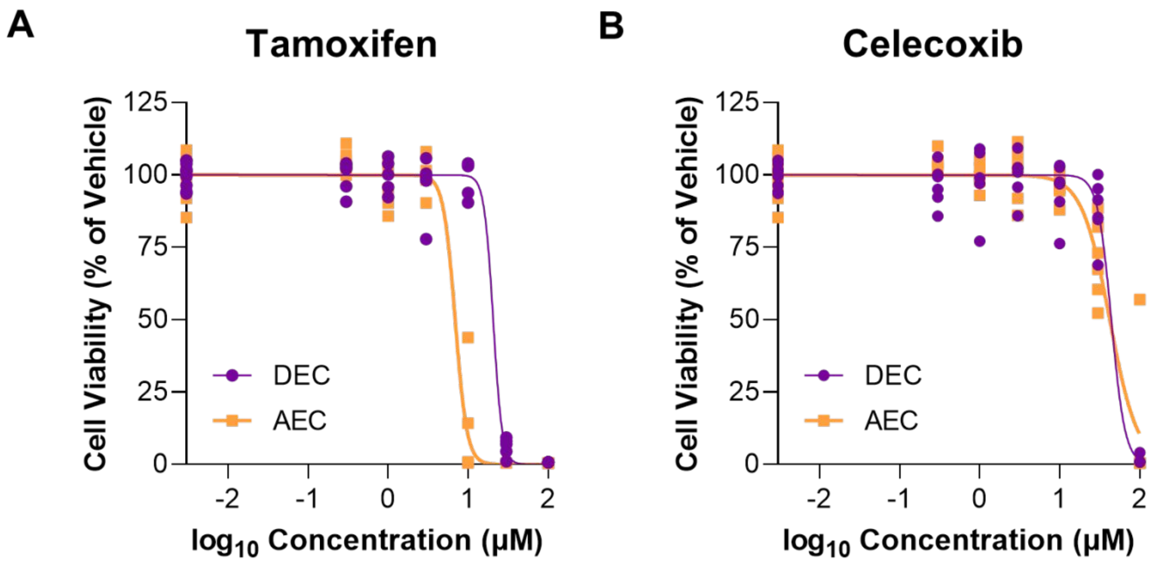

Figure S5. Normalized concentration-response effects of tamoxifen (**A**) and celecoxib (**B**) on decidual and amnion epithelial cells cultured in 96-well plates after 48 h exposure. Values are presented as mean and standard deviation (n=7-9 for vehicle controls, n=5-6 for test compounds).

Table S1. Physicochemical properties of Celecoxib, Tamoxifen, Aspirin, Sofosbuvir, and PFOA (Perfluorooctanoic acid).

Compound	Molecular Weight (g/mol)	logP (approx.)	Water Solubility	Polarity / Classification
Celecoxib (SML3031-50MG, Millipore Sigma)	381.37	~3.5	Poor	Lipophilic
Tamoxifen (T5648-1G, Sigma Aldrich)	371.51	~6.3	Very Poor	Lipophilic
Aspirin (A5376-100G, Millipore Sigma)	180.16	~1.2	High	Amphiphilic
Sofosbuvir (AMBH2D6FB19C-100MG, Millipore Sigma)	529.46	~1.6	Moderate	Hydrophilic
Perfluorooctanoic acid (171468-5G, Sigma Aldrich)	414.07	~6.3	High	Amphiphilic
